# The Experiences of Strategic Purchasing of Healthcare in Nine Middle-Income Countries: A Systematic Qualitative Review

**DOI:** 10.34172/ijhpm.2023.7352

**Published:** 2023-11-06

**Authors:** Joshua Sumankuuro, Frances Griffiths, Adam D. Koon, Witness Mapanga, Beryl Maritim, Atiya Mosam, Jane Goudge

**Affiliations:** ^1^Centre for Health Policy, School of Public Health, Faculty of Health Sciences, University of the Witwatersrand, Johannesburg, South Africa.; ^2^Department of Public Policy and Management, SD Dombo University of Business and Integrated Development Studies, Wa, Ghana.; ^3^School of Community Health, Charles Sturt University, Orange, NSW, Australia.; ^4^Warwick Medical School, University of Warwick, Coventry, UK.; ^5^Department of International Health, Johns Hopkins Bloomberg School of Public Health, Baltimore, MD, USA.; ^6^School of Health Systems and Public Health, University of Pretoria, Pretoria, South Africa.; ^7^Consortium for Advanced Research Training in Africa (CARTA), Nairobi, Kenya.; ^8^School of Public Health, University of the Witwatersrand, Johannesburg, South Africa.

**Keywords:** Strategic Purchasing, Stakeholder Capacity, Governance, Reimbursement, Middle-Income Countries, Healthcare Financing

## Abstract

**Background:** Efforts to move towards universal health coverage (UHC) aim to rebalance health financing in ways that increase efficiency, equity, and quality. Resource constraints require a shift from passive to strategic purchasing (SP). In this paper, we report on the experiences of SP in public sector health insurance schemes in nine middle-income countries to understand what extent SP has been established, the challenges and facilitators, and how it is helping countries achieve their UHC goals.

**Methods:** We conducted a systematic search to identify papers on SP. Nine countries were selected for case study analysis. We extracted data from 129 articles. We used a common framework to compare the purchasing arrangements and key features in the different schemes. The evidence was synthesised qualitatively.

**Results:** Five countries had health technology assessment (HTA) units to research what services to buy. Most schemes had reimbursement mechanisms that enabled some degree of cost control. However, we found evidenced-based changes to the reimbursement mechanisms only in Thailand and China. All countries have some form of mechanism for accreditation of health facilities, although there was considerable variation in what is done. All countries had some strategy for monitoring claims, but they vary in complexity and the extent of implementation; three countries have implemented e-claim processing enabling a greater level of monitoring. Only four countries had independent governance structures to provide oversight. We found delayed reimbursement (six countries), failure to provide services in the benefits package (four countries), and high out-of-pocket (OOP) payments in all countries except Thailand and Indonesia, suggesting the schemes were failing their members.

**Conclusion:** We recommend investment in purchaser and research capacity and a focus on strong governance, including regular engagement between the purchaser, provider and citizens, to build trusting relationships to leverage the potential of SP more fully, and expand financial protection and progress towards UHC.

## Background

 All countries need to purchase healthcare in ways that ensure resources are used effectively and efficiently; the need for healthcare will always outstrip the finances available with ever improving medical technology and so the more expensive care options that are available.^1^ Purchasing (the allocation of funds to healthcare providers for services, on behalf of identified groups or a population),^2^ requires a continuous search for the best ways to maximise health system performance. It involves deciding which interventions to purchase, how to buy them, and from which providers, how providers will be paid, at what rates and under what contractual arrangements (eg, active or strategic purchasing [SP]).^3,4^

 Given the international call for universal health coverage (UHC), many middle-income countries have started SP initiatives as part of established public insurance schemes.^2,5-7^ In this systematic qualitative review, we report on the experiences in nine middle-income countries (both lower- and upper middle-income countries) to understand what extent the activities that constitute SP have been established with public sector insurance schemes, what have been the challenges and facilitators, and to what extent SP is helping countries achieve their UHC goals.

###  What Does Strategic Purchasing Involve? 

**Figure 1:**
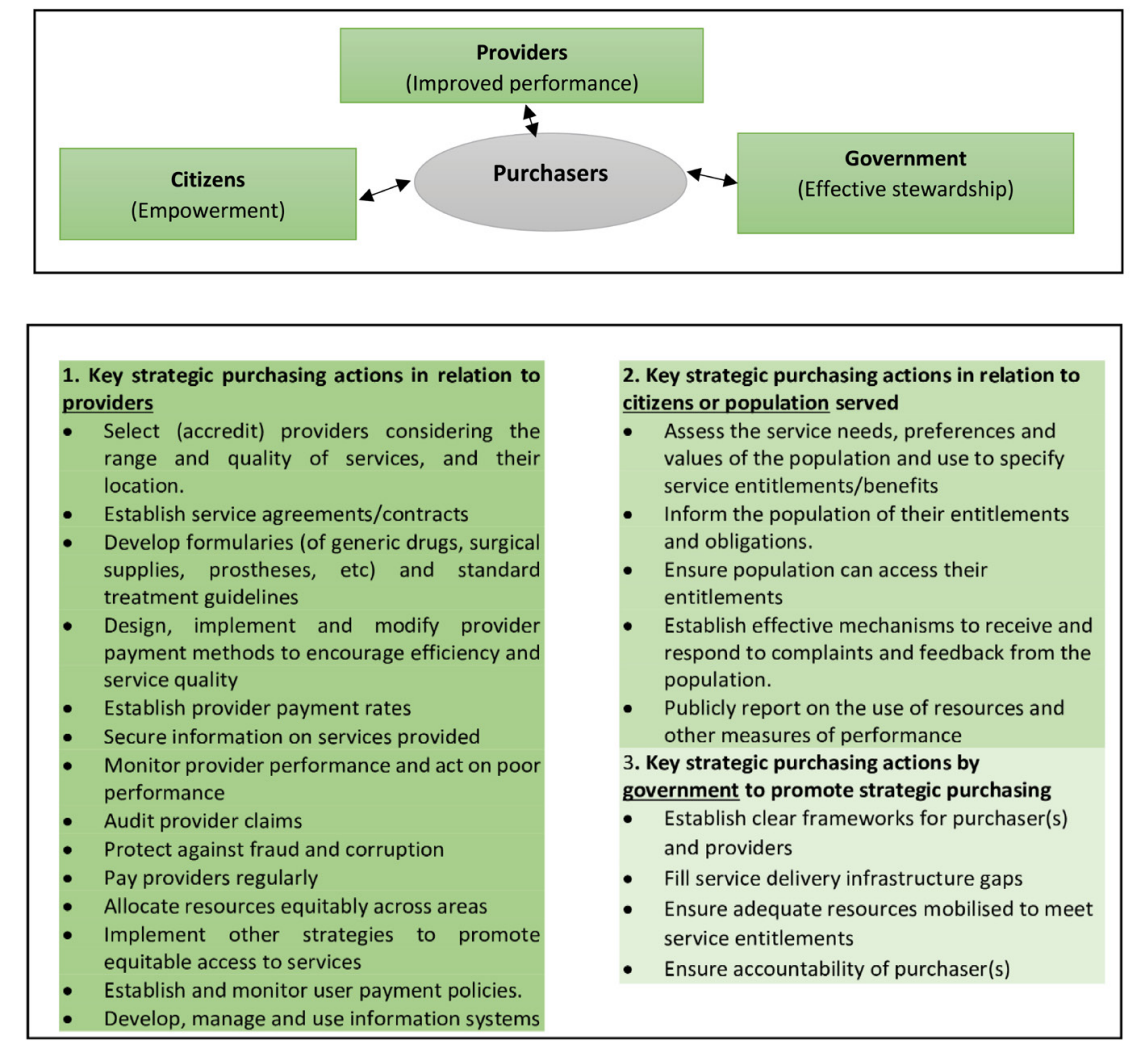
Key Actors and Their Roles in Strategic Purchasing of Healthcare. Source: From RESYST^7^ policy brief: What is strategic purchasing for health.

 SP requires the purchaser’s interaction with three key role players: the provider of healthcare services, citizens as the beneficiaries, and government as the regulator of both purchasing and provision of care. Figure 1 sets the actions associated with each key actor.

###  Universal Healthcare Coverage, Insurance, and Strategic Purchasing 

 The aim of universal healthcare coverage is to provide quality healthcare and financial protection to all people in a given country.^2,4,7,8^ Pre-payment, either through taxation or insurance, is necessary to provide financial protection.^6^ While SP can be achieved through taxation systems, insurance schemes, with their separation of purchaser and provider roles into different organisations, is where SP is more visible. The core functions of insurance include pooling resources, enrolling members, defining the benefits package, contracting and paying providers and ensuring delivery of quality care that represents value for money. SP is then an essential building block to ensure that an insurance scheme remains financially viable, and that best use is made of available funds.^2,9^ Done well, these tasks, in theory, can amount to a virtuous circle, with risk and income cross-subsidisation providing protection from catastrophic expenditure for its members, new members joining as knowledge of the scheme grows, and a benefits package that increases as more resources become available.

## Methods

**Figure 2:**
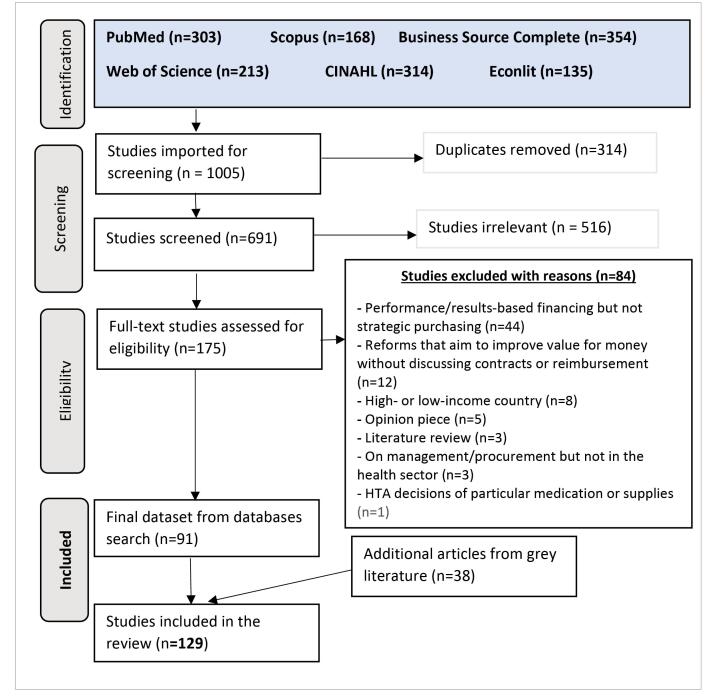
Papers Identified and Screened: PRISMA Diagram. Abbreviations: PRISMA, Preferred Reporting Items for Systematic Reviews and Meta-Analyses; HTA, Health Technology Assessment.

 We conducted a systematic search for literature in the following bibliographic databases: PubMed, CINAHL, Business Source Complete, Econlit, Web of Science, and Scopus (See Supplementary file 1, for the search syntax). We included the names of the 110 middle-income countries (as defined by the World Bank).^10^ Our search start date was 2011 as we found the rate of publications on SP increased at that time and the search was performed in November 2019. The Preferred Reporting Items for Systematic Reviews and Meta-Analyses (PRISMA) diagram presents the systematic search and screening process (Figure 2). In addition, 38 additional grey literature documents on SP in case study countries, identified through google and google scholar searches, were included (such as conference presentations and reports, from RESYST [Resilient and Responsive health systems] and the World Health Organization [WHO] Global Health Regional websites) to supplement our review.

###  Screening Strategy

 Once duplicates had been removed, 691 articles were exported to Covidence (software for managing and streamlining systematic reviews) and screened following the predefined inclusion and exclusion criteria in Box 1. Non-English articles were excluded except Spanish, which ADK is able to read.^11^ Duplicate screening was conducted at title and abstract stage with differences resolved by a third person. The full-text screening was initially done in batches of 30 in duplicate. Differences were resolved through discussion, and if necessary, the inclusion and exclusion criteria were revised to clarify any uncertainty. We continued screening in batches of 30 until no more differences of opinion arose. A total of 91 articles were found relevant to SP in healthcare in addition to the 38 retrieved from grey literature search. Thus, a total of 129 articles were include in this review.

**Box 1. Inclusion and Exclusion Criteria**

** Inclusion Criteria**

- Describe a purchasing function (either in a public national health system or specific private insurance scheme) that aims to have characteristics associated with SP, ie, desires to obtain value for money through contracts or reimbursement mechanisms.
- Includes evidence from a middle-income country as defined by the World Bank.
- Evidence reviews if they include middle-income countries.

** Exclusion Criteria**

- Those presenting evidence from either high- or low-income countries.
- Those that describe reforms that aim to improve value for money without discussing contracts or reimbursement mechanisms.
- Studies that discussed performance-based financing or results-based financing, or payment for performance.
- If studies were conference abstract, opinion piece, systematic literature reviews or grey literature.
- Studies published before 2011.
- If they discussed only revenue mobilisation, contracting, health financing or health insurance other than SP functions.
- We excluded opinion pieces and commentaries.

-------------------

Abbreviation: SP, strategic purchasing.

###  Sampling Country Case Studies

 Listing the countries in order of the number of articles on SP, we found 21 countries with one or more articles about SP. We purposively 9 selected countries to include those with the most articles whilst ensuring geographical spread. The countries were: Iran (17 articles), Nigeria (10), China (10), Mexico (7), Ghana (6), Kenya (5), Thailand (3), Vietnam (3), and Indonesia (1).

###  Search for Supplementary Articles on Healthcare Financing in Case Study Countries

 We conducted additional searches in August 2020, to find articles on healthcare financing in each case study country. We searched for relevant articles in two main databases: Scopus and PubMed. We also conducted a grey literature search. We used the search terms: ‘healthcare financing’ OR ‘health financing’ AND ‘country’ for each country included. Screening was conducted using the following criteria: the article discussed at least one public healthcare financing mechanism, addressed any element of healthcare financing (eg, benefits package, service delivery platform, reimbursement mechanisms, provider-purchaser relationships, governance systems, etc) in one or more case countries, article was published in a peer-reviewed journal or was policy document that provides details of healthcare financing reforms in the case country. Time limitation was not applied to case study searches. For each case study we identified the following number of articles: Thailand (n = 45), Nigeria (n = 55), Ghana (n = 54), China (n = 91), Iran (n = 58), Mexico (n = 41), Vietnam (n = 42), Kenya (n = 52), and Indonesia (n = 38).

###  Data Extraction and Analysis

 A data extraction template was designed and used to extract relevant information. Each team member focused on one or two case study countries. We read the supplementary articles to ensure we understood the healthcare financing system for each case study. We then focused on the SP articles. We extracted all results related to SP and made notes on each paper relating to the results to our understanding of healthcare financing in the country, and questions about SP relevant for our cross-country comparisons. We then reviewed the extractions and notes, and each team member wrote a case study summary covering the structure of healthcare financing, key health system and financing reforms, SP and the associated facilitators and challenges. We held twelve (n = 12) meetings to present the case study summaries to the rest of the team and to identify and discuss key experiences/issues, including the similarities and differences between countries’ and schemes. We used the extracted data, notes and case study summaries to finalise our analysis and write up. Samples of the data extraction files are attached (See Supplementary file 2).

###  Data Synthesis and Presentation

 While the RESYST framework emphasizes the relationships between the four sets of actors (purchasers, providers, citizens, and government), the detail provided is simply a list of specific tasks that each is responsible for executing. To develop an analytic framework within which to present the synthesis, we kept in mind the core elements of purchasing (deciding what to buy, from whom and how), as well as the importance of activities that maintain relationships between actors, and then organised the available data in the following sections with associated tables: healthcare financing country context; description of schemes (coverage, benefits etc); scheme performance; purchasing arrangements; and, governance of purchasing. (We were constrained by data availability; for example, there was data on reimbursement mechanisms but not the detail of specific contracts).

 Firstly, we compare the structure of healthcare financing in each country based on the relative financial flows (ie, public, and private prepayment spending, external funds, out of pocket, etc) to provide a contextual understanding (Table 1).

**Table 1:** Comparison of Key Health Financing Indicators Among Case Study Countries

**Indicators 2017**	**Iran**	**China**	**Mexico**	**Thailand**	**Vietnam**	**Indonesia**	**Ghana**	**Kenya**	**Nigeria**
GDP per capita ($) (2021)	3000	8840	9900	6600	3743	4223	1960	1500	2360
Economic growth over 40 years (1981-2020)	2.7	9.2	2.1	4.8	6.4	4.9	4.6	3.8	3.0
THE as a % of GDP	8.7	6.4	5.5	3.8	5.9	2.9	3.3	4.8	3.8
Government health expenditure as % of GDP	4.4	2.9	2.8	2.9	2.7	1.5	1.1	2.1	0.5
Government health expenditure as % of THE	51.3	56.7	51.5	76.1	48.6	48.4	33.5	42.7	14.2
Private health expenditure as % of THE	48.7	43.3	48.5	23.6	49.4	51.1	52.0	39.4	77.9
External resources on health as % of THE	0.4	0.00	N/A	1.5	7.5	1.9	21.3	28.3	17.5
OOP expenditure as % of THE	41.8	36.1	41.3	11.1	45.3	34.6	40.3	24.0	77.2
Government health expenditure per capita in Int$	901.4	474.8	552.2	525.8	187.4	163..8	48.5	67.2	31.36
Life expectancy	76	77	75	77	75	72	64	66	54
Maternal mortality (per 100 000 live births)	16	29	33	37	43	177	308	342	917

Abbreviations: THE, total health expenditure; GDP, gross domestic product; N/A, not available; OOP, out-of-pocket. Source: The World Bank and WHO (2021): World Development Indicators, last updated on July 30, 2021.^18^

 Secondly, we provide a description of the insurance schemes, including coverage and the benefits package, in order to compare the benefits packages (which are specified in different ways), we identified whether the packages included three particular treatments — HIV/AIDS treatment (as representative of chronic care), maternal care (including hospital delivery) because its need is widespread, and dialysis (because, its cost has a catastrophic effect on households).^12-16^ Three categories were created to represent the extent of the benefits package: (1) full package if it covers all three services, (2) partial package if it covers two of the services, and (3) limited package if only includes one of these services (Further details on membership eligibility criteria and contributions are provided in Table S1 of Supplementary file 2).

 Thirdly, we provide an indication of the schemes’ performance using levels of out-of-pocket (OOP) by insured members, whether there were reports of purchases struggling to pay for care, and providers refusing to provide care (Table 2).

**Table 2:** Outcome Measures: Out-Of-Pocket Payments and Whether Members Were Refused Care

	**Iran**	**China **	**Mexico **	**Thailand **	**Vietnam **	**Indonesia **	**Ghana **	**Kenya**	**Nigeria**
OOP by subscribers	55.0% of OOPs by IHIO, SSO and IKRF members in 2017^63^	Cash informal OOPs (called ‘red envelope’) to physicians ranges between 54.4% (2011)^64^ to 76.1% (2015)^65,66^ by UEBMI, URBMI and NCMS	Segura popular households have a lower proportion of OOP (42.95%) than households without insurance (57.05%)^67^	11% of OOP by UCS, SSS and CSMBS up to 2020^53^	Approx. 30.8% of OOP by VSS subscribers in 2020^68^ 34% OOP at commune health centres/stations 39% OOP at district hospitals^69^	18.0% OOP by JKN subscribers^70^	46.9% OOP by NHIS subscribers up to 2020^71^	29.0% OOP by NHIF subscribers up to 2017^72^	89.8% OOP FSSHIS subscribers^73^
Problems with payments	Delays reimbursing of claims	• Selective application of DRGs to selected disease conditions • No uniformity in application of the 4 payment methods for all hospitals in all provinces	• State/REPSS has difficult paying providers	Not reported	Delayed reimbursing claims	Delays in reimbursing claims	Delay in reimbursing claims, which lead to comprised quality of care, and private accredited facilities refusing to provide care for members	Delays in payments due unavailability of funds	• Delayed payment due to complex payment system • Failure of NHIS to audit payments
Providers refusing care to insured	Insured clients were not refused care	Yes, if providing care will lead to high cost beyond predefined service rate or cost ceiling	No evidence to suggest providers refuse care (The General Health Law prevents this) *Unclear what will happen under INSABI	Providers do not refuse to provide health services to subscribers	There is no evidence to suggest that providers refused care to subscribers	• No evidence of providers refusing to provide care • However, there is evidence of some hospitals not being able to offer services under the benefits package because of lack of resources^a^	Some providers especially the Catholic Health Association of Ghana refused care when NHIS delays in reimbursing claims	• Some members were refused or rationed services because of late payment of capitation and claims by NHIF • Members of the national scheme faced some discriminatory care compared to other schemes with higher reimbursement rates^72^	Patients were sometimes refused timely treatment because of delayed reimbursement of claims for previous services provided

Abbreviations: UEBMI, Urban Employee’s Basic Medical Insurance; URBMI, Urban Resident’s Basic Medical Insurance; OOP, out-of-pocket payment; IHIO, Iran Health Insurance Organisation; SSO, Social Security Organisation; IKRF, Imam Khomeini Relief Foundation; NHIS, National Health Insurance Scheme; FSSHIS, Formal Sector Social Health Insurance Scheme; NHIF, National Health Insurance Fund; UCS, Universal Coverage Scheme; JKN, Jaminan Kesehatan Nasional; CSMBS, Civil Servants’ Medical Benefits Scheme; NCMS, New Cooperative Medical Scheme; INSABI, Institute for Health and Well-being; SSS, Social Security Scheme; DRGs, diagnosis related groups; REPSS, Regimen Estatal de Proteccion Social en Salud; VSS, Vietnam Social Security. ^a^ The World Bank. Implementation completion and results report on a credit in the amount of special drawing right (SDR) 41.5 million (US$ 65.0 million equivalent) to the socialist republic of Vietnam for a central north region health support project. Hanoi: The World Bank; 2017.

 Fourthly, we compare key elements of the purchasing arrangements, including reimbursement mechanisms, presence of gate keeping rules, whether there are caps on expenditure, evidence of provider-purchaser negotiations, research capacity, and whether purchasing was decentralised or not (We judged the latter to be important in enabling purchasers to be responsive, particularly in large countries) (Table 3 and Table S1).

**Table 3:** Cross-country Comparison of Reimbursement Mechanisms and Processes

	**Iran **	**China**	**Mexico**	**Thailand**	**Indonesia **	**Vietnam **	**Ghana **	**Kenya **	**Nigeria **
PHC	FFS	Central government subsidies PHC through a line budget	Mix of historical-based funding, capitation, and activity-based funding	UCS and SSS: Capitation CSMBS: FFS	Capitation	Performance-based capitation payment at community health centres	FFS; Capitation payment method was piloted but suspended	Capitation	Capitation, FFS
Hospital	FFS and case-based payments using RVUs^a^ or RBRVUs for outpatient and in-patient services, respectively	• Reimbursement mechanisms differ by province • DRGs in 20 of 32 provinces • Otherwise scale payment, FFS, capitation (in-patient and out-patient)	Line budgets, case-based payments, FFS	• DRGs for in-patient services • FFS for some services with high levels needs of the population such as cataract surgery, hip replacement therapy, etc	• DRGs is used in paying in-patient services • FFS payments • In-patient “hotel” care funded through a per-diem-payment with co-payments	• Case-based group (a type of DRG) • FFS payment was applied to only small portions of care	DRGs	• Case based payment for bundled care eg, maternity, renal and surgical care • FFS for radiology (MRI and CT scan capped)	• FFS based on authorised referrals • Per case payment
Medicine	FFS	FFS	FFS	FFS	FFS	FFS	FFS	• Outpatient – including in capitation • Inpatient • FFS	FFS
Purchaser-provider negotiation	• No platform for negotiations among stakeholders • Government independently determined the tariffs for purchaser organisations	• No evidence of negotiation • Pricing was determined by the National Health Development Research Centre	• Fees for physicians are negotiated through short-term contracts • Drugs are negotiated with suppliers at central level by a commission with diverse representation	Purchaser, provider, and citizens were engaged in policy initiatives and negotiations, ie, policy-making, design of health benefits package, budgetary processes	• No evidence of negotiations between purchasers and providers in fixing INA-CBG rates, caps of certain health services, etc • BJPS and the MoH determined package prices	• The MoH responsible for setting policy for both public and private providers including benefits package and setting reimbursement prices and co-payments • No negotiations with beneficiaries	There were purchaser-provider negotiations. However, these were inadequate which led to stalling of the roll out of the capitation payment methods	NHIF board was mandated by law to determine the rates and claims in consultation with private and public providers	• Expert committee with representatives of HMOs, providers, the NHIS, civil society organisations, academia and the Federal MoH defines benefits packages • Evidence from actuarial study was used to determine rates for capitation and FFS
Budget support to public providers	• Government allocates funds to public health services, although, inadequate • Salary and incentives paid to medical university staff based on the medical procedure or speciality	Government subsidises public healthcare, although the subsidy level varied across the regions	Under Seguro Popular, providers were issued short-term contracts without benefits	There are adequate and regular budgetary allocations for public providers	Budgetary allocations are made to public providers	• There is annual budgetary support to public providers • Government pays salaries of public providers	Annual budgetary allocation to public health providers	• Public facilities are government allocated budgets and salaries • Casuals and support staff in public facilities are paid from user fees^b^	• The MoH pays staff salaries, mainly for tertiary hospitals • Budget support is inadequate for primary care

Abbreviations: FFS, fee for service; DRG, diagnosis related groups; UCS, Universal coverage scheme; SSS, social security scheme; CSMBS, Civil Servants’ Medical Benefits Scheme; MoH, Ministry of health; BJPS, Badan Penyelenggara Jaminan Sosial; PHC, primary healthcare; RVUs, relative value units; RBRVUs, resource-based relative value units; MRI, magnetic resonance imaging; CT, computerized tomography; INA-CBG, Indonesian-Case Based Group; NHIF, National Health Insurance Fund; HMOs, health maintenance organizations; NHIS, National Health Insurance Scheme. ^a^RVUs is the real monetary value for health services. ^b^“user fees” is a charge imposed by the government for the primary purpose of covering the cost of providing a service, directly raising funds from the people who benefit from the care or service being provided.

 Fifthly, we described the extent and type of governance because of its impact on SP (Table 4). We used the WHO framework on governance for SP to compare the case countries.^4^ The framework has three key areas: setting directions, coordination and alignment, and legal provisions and regulations. We used the following indicators for comparison: (1) the existence a 5- or 10-year policy/strategy document (setting direction); (2) existence of governance body (coordination and alignment); and (3) a legal framework (legal provisions and regulations). We supplemented this with evidence on accreditation, monitoring of the claims, evidence of corruption and strategies to reduce it, patient engagement and patient feedback channels.

**Table 4:** Cross-country Comparison of Strategic Purchasing Governance

	**Iran **	**China**	**Mexico**	**Thailand**	**Indonesia **	**Vietnam **	**Ghana **	**Kenya **	**Nigeria **
Governance bodies of insurer/purchaser	All 3 public insurance organisations (ie, IHIO, SSO, IKRF) and the national health security organisations has structures eg, HCHI	NHC, MoHRSS, and MoH	MoH, the COFEPRIS and the GHC, the REPSS, SHS	UCS, SSS, and CSMBS have clearly defined governance structures and their interrelationships with providers, NHSO and comptroller and accountant general	There exist governance structures for JKN, BPJS, and the provider	• The Department of Health Insurance and the VSS • There are provincial people’s committees that monitor revenue collection and payments at provincial level	NHIA and NHIS has clearly mapped governance structures	NHIF has a governance structure and is regulated by the National Hospital Insurance Fund Act of 1998	• The NHIS has formal structures for managing providers and HMOs • The NHIS runs the FSSHIS and statutorily oversees the HMOs
Existence of provider governance body	MoHME	NHC, MoHRSS, the MoH of China supported by the various local and provincial government department	Multiple. • The primary one is Consejo de Salubridad General (GHC). Council has Executive Board made of heads of public institutions • Other councils, commissions and committees are represented on GHC^a^ • MoH • The COFEPRIS • The REPSS and SHS	MOPH, have clearly defined governance structures and their interrelationships with providers, NHSO and comptroller and accountant general	• Indonesian MoH • DJSN, the National Social Security Board. DJSN comprises both government officials, community members, and representatives of employee and employer associations	• MoH, The Department of Health Insurance, VSS Agency, Social Affairs Committee of Vietnam National Assembly • There are Provincial People’s Committees that monitor revenue collection and payments at provincial level	MoH, NHIA	• MoH, NHIF, Kenya Medical Practitioners and Dentists Board • NHIF has a governance structure and is regulated by the National Hospital Insurance Fund Act of 1998	• SMoH, HMOs • There are laws guiding the activities of government actors (eg, MoH), HMOs and providers • The NHIS has formal structures for managing providers and HMOs • The NHIS runs the FSSHIS and statutorily oversees the HMOs
Accreditation and monitoring of quality of care	• The MoHME assesses and accredits providers • No monitoring of providers’ compliance with clinical guidelines	• Local and international bodies accredit health facilities • Providers are accredited by JCI	• Accreditation is mandatory for Seguro Popular providers, but not IMSS or ISSSTE • Accredited by Specialty Councils and MoH – COFEPRIS/DGCES to enable funding by CNPSS^b^ • ISSSTE has developed a set of 44 quality and efficiency indicators for its hospitals	Thai HAI provides accreditation to providers using the ISQua	• The Hospital Accreditation Committee assesses facilities every 3 years using ISQua standards • BPJS-Health conducts onsite supervision of providers and provides technical support to public providers • BPJS-Health Office conducts regular public reporting on providers; showcases each provider's performance under the commitment-based capitation^c^ payment policy (KBK) payment system and benchmark each provider compared to their peers	The MoH accredits service providers^100^	Ghana Accreditation Board in collaboration with the MoH assesses and grants accreditation to private providers. However, public providers receive automatic accreditation	NHIF has a benefits and quality assurance management committee for monitoring the quality of services offered by providers. However, this was rarely done to inadequate capacity of NHIF to perform this function	• HMOs are accredited and registered by the NHIS to purchase healthcare services from providers on behalf of the NHIS. • NIHS rarely reviews/reaccredits HMOs and providers (lack of resources) • HMOs conducts quarterly review of providers to ensure provision of quality care
Monitoring claims	No clearly established monitoring systems in place for monitoring claims	Have introduced electronic health records and e-claim processing	There are information systems for tracking monitoring quality of care	• NHSO had a thorough system of medical audits to prevent fraud. Use of global budgets prevented ‘DRG-Creep’ • Created unique identification number for each medicine to enable monitoring of use by prescribers	• The district health offices conduct monthly monitoring of providers to track targets • However, on-site monitoring and technical support to providers is inconsistent • Evidence of fraud in provider claims processing • Although evidence of active monitoring and enforcement of capitation payment rules (eg, a private primary provider’s contract was retracted/terminated for violation of terms)	• In 2015, VSS set up electronic claims management system and this provided insured persons with a smart card • In 2017 – 97% of service providers had access via a portal to the VSS claims management system, and 60% connected daily	• There are provider monitoring units in all NHIS offices • Claim processing units are established in all health facilities to ensure proper completion of claims and adherence of providers to defined costing guidelines • Auditing of historical claims data to identify fraud • Claims centre has been able to detect fraud in claim processing	NHIF responded to the rise of fraudulent claims by employing medically trained personnel to review medical claims before payment	• HMOs compile and send patient encounter data quarterly to NHIS • Facilities do not always send the data to HMO • No systematic approach and large variations in what is done and how often • NHIS is meant to audit payments to ensure timely reimbursement of claims, however, this is not done^34^
Evidence of corruption	• “Under table” payments for health services • Evidence of repeated visits to particular physicians in a short time • Dispensing prescriptions at a particular pharmacy suggests collusion between physician and pharmacy over prescriptions, inflates prescription claims by over 25%	No evidence identified	• Mostly tied to state-level purchases of medicines and human resources • There is also a political side to corruption. For example, a former governor of Tabasco State was sent to jail in 2013 for mishandling funds for Seguro Popular. Other states have faced similar allegations	Providers falsified DRGs called “DRG-Creep” where up-coding of diagnosis in favour of higher DRG weights	• Over prescriptions or clients lying to prescribers on specific health conditions, and afterwards go to sell those medicines at “underground market” • Professionals falsifying services that were never provided • Repeated submission of claims for a similar service • Changing the dates/medicinal records on patient records • Utilisation of unlicensed staff to provide substandard care	• Civic organisations have documented corruption in Vietnam Besides documented inequalities due to misuse of resources and differences in services provided in provinces,^101^ there was no further evidence of corruption	There were cases of corruption such as inflation of claims, overbilling of medicines, inappropriate use of tariff, duplication of claims, lack of diagnostic evidence to back claims, absence of linkage between treatment and diagnosis, and treatment outside benefits package	There were reports of corruption in the accreditation process and the processing of claims	• Many HMOs are owned by financially and politically affluent citizens, some of whom serve as members of the NHIS governing council • Currently no legislation that prohibit this arrangement
Strategies to reduce corruption	• In the fifth development plan (2011-2015), teaching hospitals were granted autonomy to ensure staff satisfaction and decrease in provider fraud • A new payment system was introduced, to change the costs of clinical services and balance hospitals’ revenues and expenses	The National Health and Family Planning Commission promulgated circulars that established “prohibitions” on corrupt practices among providers, and “blacklisted" system on pharmaceutical and medical device providers	• Adjustment of regulatory framework with CNPSS managing resources and times of transfer of resources throughout the different levels (Federal, state, REPSS) • The CNPSS and the REPSS had increased accountability for Segura Popular through new accounts created at the Federal Treasury, and new sanctions established	NHSO manages malpractice by applying a global budget on top of the DRG system and a thorough system of medical audit administered	• Public Research Anti-Corruption Clearing House and the Corruption Eradication Commission established in 2015 to prevent provider fraud • An e-tendering policy for drugs and supplies was introduced in 2014 to expedite contractual arrangements and reduce corruption	• In 2015, the government embarked on a project to enable electronic claims management • Provided insured persons with a smart card • By 2017 – 97% of service providers had access via a portal to the VSS claims management system, and 60% connected daily	• Implementation of e-claim submission/processing platform • Introduction of clinical audit and historical auditing of claims through the claim processing centres	• NHIF employed staff with medical training to review claims made by providers • There are also recent plans to cede the function of accreditation to the MoH	No evidence of any
Channels for feedback from members	There is a hotline “1690” to report informal payments and report patient complaints about providers	Patients can lodge complaints at the respective hospitals, or through complaint letters	• Members can lodge complaints through the CONAMED • The CNDH intervenes in high-profile cases • A Patient’s Charter was published by the CONAMED and replicated in human rights guidelines	• A 24-hour call centre service (code “1330”) was established to create public awareness about members entitlements and for patients’ complaints and resolutions • The call centre was established for all three schemes, although, evaluations showed underutilisation	• BPJN Kesehatan also mandates all providers to have a patient complaints and resolution box • Complaints were received through the box and monthly meetings by facility management to resolve the issues • Customers could also make complaints through the customer relations office	The MoH has a dedicated division within the Department of Health Insurance that deals with issues reported by members	NHIS member complaints hotline was established to monitor provider-patient behaviours/relationships	A toll-free line and email address is indicated in their NHIF website and publicity materials but there were reports that the phone number is not functioning	• HMOS are mandated to conduct quarterly seminars with beneficiaries; few people engage as communication channels are not clear • HMOs hold forums to inform people of their benefits and entitlements, but there is limited awareness amongst users of the forums • A complaints system exists but is not fully implemented

Abbreviations: IHIO, Iran Health Insurance Organisation; SSO, Social Security Organisation; IKRF, Imam Khomeini Relief Foundation; NHSO, National Health Security Office; HCHI, High Council of Health Insurance; NHC, National Health Commission; MoHRSS, Ministry of Human Resources and Social Security; MoHME, Ministry of Health and Medical Education; COFEPRIS, Federal Commission for the Protection Against Sanitary Risks; REPSS, Regimen Estatal de Proteccion Social en Salud; CSMBS, Civil Servants’ Medical Benefits Scheme; SSS, Social Security Scheme; UCS, Universal Coverage Scheme; BPJS, Badan Penyelenggara Jaminan Sosial; JKN, Jaminan Kesehatan Nasional; VSS, Vietnam Social Security; NHIA, National Health Insurance Authority; NHIF, National Health Insurance Fund; HMO, health maintenance organization; FSSHIS, Formal Sector Social Health Insurance Scheme; GHC, General Health Council; DJSN, Dewan Jaminan Sosial Nasional; SMoH, State Ministry of Health; JCI, Joint Commission International; IMSS, Instituto Mexicano del Seguro Social; ISSSTE, Institute for Social Security and Services for State Employees; DGCES, General Directorate for Health Quality and Education; CNPSS, National Commission for Social Protection in Health; ISQua, International Standards for Quality in Healthcare; HAI, Health Accreditation Institute; KBK, Kapitasi Berbasis Komitmen; CNDH, National Commission for Human Rights; DRGs, diagnosis related groups; REPSS, Regimen Estatal de Proteccion Social en Salud; MoH, Ministry of Health; SHS, State Health Secretariats. ^a^Councils (against Addictions, for Accident Prevention, and for the Prevention and Control of HIV/AIDS), Commissions (Bioethics, Human Genome, Occupational Health and Safety, Human Resources Development, and Health Research), Committees (Oral Health, Care for the Aging, and Epidemiological Surveillance) and the Reproductive Health Group. ^b^Under INSABI, plans are underway to transfer accreditation to the GHC. ^c^Under the commitment-based capitation policy (2016) BPJS-Health employs indicators of staff commitment to decide on the capitation grant percentage to allocate to primary health provider.

## Results

###  Healthcare Financing Context in the Case Study Countries

 In Table 1 we compare the case study countries’ health financing indicators (in 2017), life expectancy and maternal mortality as indicators of health system performance, as well as their average economic growth rates for the past 40 years (1981-2020) (The order of the countries in Table 2 is based on their maternal mortality rate).

 Government expenditure on health was between 2%-3% of gross domestic product (GDP) for five countries (China, Mexico, Thailand, Vietnam, and Kenya) and below 2% in three countries (Indonesia, Ghana, and Nigeria); only in Iran was it above 4% (Evidence suggests that government expenditure needs to be above 5% of GDP to achieve UHC).^17^ Public and private expenditure amounted to roughly equal shares of total health expenditure in 6 countries, with the exception of Thailand (76%/23% split), Ghana (33%/52% split), and Nigeria (14%/77%). External donor resources were significant in Ghana, Kenya, and Nigeria (17%-28%). Out of pocket expenditure as percentage of total health expenditure was between 30%-45% of total expenditure in all the case study countries, except for Thailand (11%), Kenya (24%), and Nigeria (77%).^18^

 Despite a GDP per capita of US$ 3000, and an average growth rate of 2.7% over the last 40 years, Iran had the highest government expenditure on healthcare as percentage of GDP (4.4%) as well as in terms of international dollars (purchasing power parity $); it was also the best performing with the lowest maternal mortality ratio (MMR) of 16/100 000 live births, and a life expectancy of 76 years, although OOP payments are high at 41%.^18^ Thailand, with its high government share of health expenditure (76.1%), manages to keep out of pocket expenditure low (11%), although its MMR is still double that of Iran. Nigeria is the worst performing country, with MMR of over 900, OOP over 70% and the government share expenditure of total expenditure at 14% (See Table 1).

###  Coverage and Benefit Packages Provided by Schemes

####  Insurance Coverage

 Five countries had more than 70% population (including rural population) covered by public sector insurance schemes [Thailand (98.5%), China (96.9%), Iran (90%), Vietnam (87%), and Mexico (85%)]^15,19-21^ (Table S1). Indonesia, Ghana, Kenya, and Nigeria had less than 50% of the population covered by state insurance schemes with Nigeria having only 5% coverage of the population by the Formal Sector Social Health Insurance Scheme (FSSHIS).^22-25^ While most schemes’ membership were primarily formal sector employees (Kenya, Indonesia, and Nigeria),^26-31^ some governments provided insurance coverage for the poor in social health insurance (SHI) schemes such as the Universal Coverage Scheme (UCS) in Thailand, Imam Khomeini Relief Foundation (IKRF) in Iran, Urban Employee’s Basic Medical Insurance (UEBMI) in Chine, National Health Insurance Fund (NHIF) in Kenya, and SHI in Vietnam.^32-38^

####  Health Benefits Package 

 Six of the countries (Iran, Thailand, Indonesia, Ghana, Vietnam, and Kenya) had a comprehensive benefits package by our categorisation, including maternity services with inpatient delivery, HIV/AIDS treatment, and at least part of the costs of dialysis in their benefits package.^33,39-45^ China, Mexico, and Nigeria’s schemes covered only maternity services, but neither HIV treatment nor dialysis^14,45-48^ (See Table S1).

###  Performance

 We found evidence of delays in payments of providers in six of the countries (Iran, Mexico, Vietnam, Ghana, Kenya, and Nigeria). We found reports that providers in Nigeria, Vietnam, and Ghana failed to honour essential services contained in the package, often due to delays in payments by the purchaser.^34,39,49-51^ Christian Health Association of Ghana returned to full OOPs when national health insurance authority (NHIA) delayed reimbursing their facilities for services provided.^52^ In Indonesia there was evidence of some hospitals not being able to offer services under the benefits package because of lack of resources.^29,51^

 In Thailand of pocket payments (OOPs) were below 15% of total health expenditure, suggesting catastrophic expenditure for households would be minimal.^53^ In Indonesia, OOP was also relatively low at 18%; Kenya 29% and Vietnam 30%-39% were the next lowest.

 Only in Thailand, Mexico, Vietnam, and Indonesia was the OOP of members reported to be lower than, or equal, to the national OOP suggesting the scheme was achieving its goal of protecting members to some degree from catastrophic expenditure (Tables 1 and 4). (In Thailand OOP by members and nationally were the same, as coverage is close to 100%). In other countries OOP by members was reported to be higher than the national figure.

 While OOP is complex to measure and therefore there is often considerable variation in its estimates, these figures suggest that the insurance schemes, other than Thailand and Indonesia, were failing to protect members against catastrophic expenditure, and that members were wealthier in comparison to the uninsured and so able to pay out of pocket when the insurance scheme fails them.^29,39,49,51^

###  Purchasing Arrangements

####  Reimbursement Mechanisms

 For primary care, the case study countries predominately used capitation as the reimbursement mechanisms, occasionally with performance-based elements (China and Indonesia),^54,55^ and fee for service (FFS) for some specific services, or in specific schemes (Thailand).^54^ Indonesia expanded the use of capitation into commitment-based capitation (using indicators to assess health facility staff commitment to work), along with increased monitoring by BPJS (Badan Penyelenggara Jaminan Sosial)-Health. The introduction of capitation and gate-keeping led to a reduction in demand for hospital care.^54^ The exceptions were Ghana and Iran where FFS reimbursement is used.^43^ Capitation has been piloted in Ghana. As mentioned above, after protests by both providers and members, partly due to inadequate stakeholder engagement, and insufficient preparation and research, the capitation payment mechanism was abandoned.^43,46,56^

 Most countries use a case-based payment of varying sophistication for hospital reimbursement, ie, Nigeria, China, Mexico, and Thailand use diagnosis related groups (DRGs),^8,57-59^ while the others used simpler forms, in combination with FFS for medication and for some specific procedures. Only Vietnam used a per-diem payment.^60^ China piloted its DRGs before scaling-up, but the implementation was negatively affected by insufficient information system capacity and lack of unified disease classifications. The pilots led to the adoption of mixed provider payment methods,^55,57^ with hospitals in some provinces allowed to choose FFS for older patients and those with complications, leading presumably to selection of the more profitable option by providers.^57^ Vietnam, Nigeria, and Kenya are using FFS for hospital care and had no process to reform provider payment methods.

 Two countries have attempted to increase the supply of particular services by switching to FFS. Thailand changed the reimbursement methods for HIV and cataracts from capitation to FFS in order to increase supply,^8,15^ and China made specific payments for TB services, and paid for patients’ transport and a subsistence allowance in order to ensure better TB outcomes.^61,62^

 Governments in all nine countries either paid salaries of staff in public facilities or provided other budgetary support (Table 2).

####  Gate Keeping

 Gate keeping has been implemented in some countries (ie, Vietnam, Ghana, Iran, Indonesia, and Thailand (except the Civil Servants’ Medical Benefits Scheme [CSMBS]), although implementation varied across countries.^8,74-76^ Ghana, Vietnam, and Thailand had a defined list of health services for which referral was permitted under the gate keeping system.^13,15,76^ In Indonesia and Mexico, there were no clearly defined services or the cost that are covered during the referral. In the literature reviewed there was no mention of a gate keeping system in Nigeria, Kenya, and China (Table 2).

####  Budgetary Caps on Expenditure

 Although reimbursement rates for particular services are part of each contract, purchasers need to control overall expenditure, to ensure that it does not exceed the scheme’s income, and so its sustainability. This can be done by setting an overall limit and adjusting the payments rates per service, should the quantity of services push threaten to push expenditure above the limit (for example UCS in Thailand).^19^ Alternatively, expenditure limits per person can be set; this is used in China, Indonesia, and for certain services in Kenya.^54,77-81^ There is no cap on overall expenditure for the CSMBS in Thailand, and in Iran, Mexico, Vietnam, Ghana, or Nigeria we found no mention of caps on expenditure (Table 2).

####  Provider-Purchaser Engagement

 Provider-purchaser negotiations are important in determining prices, the affordability of services and the sustainability of a scheme. Purchaser-provider engagement can enable sharing relevant information and building relationships based on trust and collaboration. Lack or limited engagement with providers is more likely to encourage provider opportunism to meet income targets.^19,33,82^

 In two countries (Indonesia and Thailand) the purchaser has effective means of engaging with providers,^8,83^ with well-structured forums. In Thailand, providers were involved in the national health security board of UCS. The National Health Security Office (NHSO) uses its substantial purchasing power to negotiate for lower prices for some selected high-cost medicines and medical devices, leading to cost savings, increased affordability, and access to essential services.^15,84^ For example, when cataract services were on high demand, the NHSO used its central bargaining capacity to negotiate an affordable price for soft lens for providers. Therefore, hospitals could then choose to reimburse on an agreed rate or to use the lens supplied by the NHSO-negotiated vendors.^8,15,84^

 Indonesia has reaped substantial benefits from engaging with providers. Performance indicators, on which price revisions and capitation payments were benchmarked, enabled the purchaser to hold primary health providers accountable and minimised opportunistic behaviour (such as using lower cadre of staff despite regulations), despite the administrative burden associated with its execution.^83,85^ This was possible through the PPJK agency (Perusahaan Pengurusan Jasa Kepabeanan-health Insurance Directorate) under the Ministry of Health (MoH) to evaluate and calculate prices, simulated with expected revenues.^54^

 In six countries (Ghana, Nigeria, Vietnam, Kenya, Mexico, and China), we found limited evidence of active engagement, and prices, benefits, and modes of payments are often fixed by either boards or committees (Kenya, Mexico, and Ghana) and the health ministries (Vietnam). In Kenya, the private sector, represented in the NHIF governing board, had a strong voice, and influence, leading to a ‘purchaser capture,’ demonstrated by the favourable reimbursement rates and terms extended to private health facilities.^86^

 Inadequate provider-purchaser engagement and protests have led to Ghana’s capitation policy being suspended; the policy was decided at the level of “elite stakeholders.”^43^ However, patients needed to know what interventions were included in the benefits package.^41,87^ Health professional associations and providers need to assess the likely effect on service delivery, the services covered in health benefits package, and their income levels.^43^ Likewise, in Iran the Ministry of Health and Medical Education (MoHME) independently determined the prices for benefits and the revision of relative value units has faced multiple challenges because of lack of active negotiations with relevant stakeholders.^19,88^

 Purchaser-provider tensions were a key contributor to the unravelling of Mexico’s decentralised, SP units within *Seguro Popular* in favour of a centralized system under the Institute for Health and Well-being (INSABI). Decentralised purchasing units struggled to negotiate effectively with centralised and highly influential medical unions. This led to increased contracting which distorted state-level fund allocations (eg, some states were spending nearly 70% of costs on contracts, causing purchasers to introduce a 40% cap). Efforts to “regularise” contracts significantly increased costs (eg, by over 30% in one year). However, better contracts did not produce better performance from providers. This suggests that federal-state coordination was poor, and oversight of purchaser provider negotiations was insufficient.^89,90^ However, INSABI, the new centralised purchasing body, emerged without adequate stakeholder consultations^91^ (Table 2).

####  Research

 Five countries (Thailand, China, Indonesia, Vietnam, and Mexico) have established health technology assessment (HTA) units, key for deciding what services and technologies to purchase.^54,92-94^ Ghana has some internal capacity within government to conduct research and has initiated the process to establish HTA. In Indonesia the National Basic Health Research Unit, established in 2013, has improved the availability and quality of data on which to make purchasing decisions. The government has also started to collect village level data to guide planning and policy decision-making.^83^ Nigeria, Kenya, and Iran had little internal capacity, and relied on publications produced by university academics (Table 2).

####  Decentralisation

 Although decentralization of healthcare service delivery has been accepted globally as a means to improve the responsiveness of the health system, decentralisation of the purchasing function is less common. In three of our case study countries (Ghana, Iran, and Vietnam), purchasing was carried out at national level.^19,22,33,95-97^ Three countries (Thailand, Indonesia, and China) have decentralised the purchasing function to the local level.^84,98^

 Thailand implemented decentralisation by increasing budgetary allocation from 9% to 26% to local governments between 1999 and 2012, to increase their purchasing capacity.^8^ However, the government’s action plan has not been fully implemented (such as the involvement of community committees in purchasing) due to a frequent change in governments. The largest public health insurance scheme in China — New Cooperative Medical Scheme has been decentralised to the state level, which give local governments vast autonomy in system design, leading to varying degrees of local government subsidies for premiums, levels of coinsurance and deductibles and reimbursement procedures.^98^ Given that Indonesia’s sizeable population, spread over many islands with diverse ethnic and religious groupings, the country has adopted high levels of decentralisation, with district level schemes having considerable autonomy in terms of scheme design and purchasing of healthcare.^35,99^

 Under the *Seguro Popular* in Mexico, the central government provided funds to state-level autonomous purchasing units called Regimen Estatal de Proteccion Social en Salud (REPSS).^102^ The REPSS were designed to exist outside of the state health agencies, in order to separate financing from provision, as a mechanism to improve the efficiency and quality of service delivery.^102^ However, recent reforms have eliminated REPSS, effectively (re)centralising purchasing to a national level under INSABI^91,102^ (Table 2).

###  Governance of Purchasing

####  Policy and Legal Frameworks, Oversight Bodies

 Indonesia, Iran, Vietnam, Kenya, and Thailand had policy frameworks guiding health system and SP reforms. Specifically, Thailand began health reforms in 1942 with an evidence-based National Economic and Social Development Plan (NESDP), which contained six separate reforms.^95^ Indonesia had a detailed policy document that provided the sequence for health reforms starting from 1945. Iran transitioned through five sequential reforms from 1964 to the 2014 Health Transformation Plan.^19^ Four countries have no explicit policy framework (Ghana, Nigeria, Kenya, and Vietnam).

 Only four countries had independent governance structures to provide oversight. In Kenya, there is a 12-member NHIF board,^38^ 31-member UCS board with diverse membership (including non-governmental and civil society organisations in health, and members) in Thailand,^15,103^ Ghana’s NHIA has a governing council,^22^ and BPJS in Indonesia has a 2-member board of director and commissioner.^104^ In Mexico, the General Health Council, a collective decision-making body composed of various stakeholders [representatives from the National Commission for Social Protection in Health (CNPSS), MoH, REPSS], defined and updated the package of high-cost interventions and assists with provider accreditation.^102^ Many of these boards have patient and private provider representation.^8,105^ However, the management board members (ie, CSMBS) in Thailand,^15^ Ghana and Kenya perform functions that were unrelated to their expertise.^33,38,105^ Moreover, large boards (eg, 31 for national health security board of UCS in Thailand) can also delay decision-making.^15^

 All case study countries had legal and regulatory frameworks establishing the schemes and setting directions for purchasing and service provision, as well as established institutions for implementing SP and associated reforms.^21,33,38,91,95,106-109^ However, diversities exist in how they were constituted and operationalised, for example in Nigeria, although the NHIS was overseeing the activities of health maintenance organizations (HMOs), the activities required of the HMOs were not properly defined, leaving lapses for opportunism.^34,108^

 Only four countries had functioning/integrated information management system, including e-databases of clients and e-claims/tendering processes (China, Thailand, partly implemented in Ghana, and Mexico).

####  Provider Accreditation 

 All the case study countries have some form of mechanism for accreditation of health facilities. Three countries use standards provided by international bodies (Thailand, Indonesia, and China), such as the International Society for Quality in Health Care (ISQua) (Thailand, Indonesia) and Joint Commission International Standards (China).^104,110,111^ For most countries there were reports of either infrequent assessments, with considerable variation in terms of what is done in which province or state (Nigeria, China, Vietnam, Mexico, Ghana, and Iran). Key challenges reported were inadequate and fraudulent assessment and ranking of providers (Iran and Indonesia),^104,107,111^ as well as inadequate and non-strict criteria for credentialling providers (Ghana, Kenya, Indonesia, and Iran).^40,104,111-113^ In some countries, public providers are given automatic accreditation (Ghana and Kenya).^113,114^

####  Monitoring Claims and Services

 Monitoring of claims is important to identify fraud. All case study countries had instituted some strategy for monitoring claims, but they vary in complexity and the extent to which they were implemented. Three countries (Ghana, Vietnam, and Thailand) have implemented e-claim processing to reduce processing time and ensure timely payment of providers (only some health facilities in Ghana). Ghana and Indonesia have established paper-based claim processing units in all health facilities.

 Electronic health records are a significant milestone in being able to check for service over-supply (Ghana, China, Indonesia, and Thailand). An integrated health information management system has been developed in China (One Health Information Management) with unique patient and prescriber identification numbers.^115,116^ Thailand uses its electronic system to track claims of accredited providers.^15^ For Ghana, the system allows for auditing of historical claims data.^22,117^

 Clinical audits of the services covered in claims were conducted in Ghana, Thailand, China, and Mexico.^15,22,118,119^ In Mexico, *Instituto Mexicano del Seguro Social* scheme tracks and audits providers for services provided under the SHI.^91^ Indonesia utilises a taskforce against fraud to clampdown retrospectively on financial wastage, through on-site monitoring of community health centres and district heath offices at given time periods.^120^ Provider performance is publicly showcased through quarterly hearings. These strategies have identified ‘under the table’ payments from patients, excessive treatment, and fraudulent use of insurance cards by non-members.^121^

 Although HMOs in Nigeria are mandated by the law to check claims sent from accredited health facilities on a quarterly basis, this is not often done, thereby creating variations in claim amounts versus services provided.^34,108^ In Iran, the MoHME was responsible for monitoring providers and purchaser organisations for compliance with clinical guidelines and audit regulations,^122^ however, this is not done.

 Key explanations for the failure in countries where monitoring was inadequate were limited capacity within NHIF (Kenya),^123^ influence of politicians and other beneficiaries of the dysfunctional system (Nigeria).^124^

####  Patient Engagement 

 In two countries, patients’ rights are enshrined in law (Mexico, China), and there are channels for patients’ complaints in six countries (Ghana, Thailand, Mexico, Nigeria, China, and Kenya). In five countries, health insurance schemes have hotlines for patients to report complaints (Thailand, Mexico, Ghana, Iran, and Indonesia).^15,22,91,122^ In Mexico, complaints can be lodged at complaints units or health facility user orientation offices, which are part of the State Health Secretariat.^91^ In Indonesia there are two mechanisms for patients to voice concerns: patients’ complaints and resolution box (paper-based system) and the customer relations office of the scheme (either in person or by phone), where monthly meetings are held to resolve clients’ issues.^120,125^ However, across countries, the channels functioned poorly, and information gained from patients are not fed back into the system to improve things. Moreover, patients’ awareness of their rights, their entitlements and how to access the benefits was poor.^120,121^

####  Corruption and Strategies to Reduce it

 We found reports of corruption in eight countries (Iran, Mexico, Thailand, Vietnam, Indonesia, Ghana, Kenya, and Nigeria). Typical of these were:

- Over prescriptions (Iran, Indonesia), inflation of prices of medicines (Mexico, Ghana),^91,126,127^ collusion (physicians had certain patients make repeated visits to them within short intervals, and those patients were directed to a particular high-cost pharmacies) between dispensaries and patients (Iran).^111,122,128^
- Falsification of DRGs coding (called “DRG-Creep’) (Thailand),^15^ inflation of claims (Ghana),^126^ inappropriate use of tariffs (Ghana),^127^ claiming services that were not provided (Indonesia, Ghana),^126,127^ and ‘under table’ payments (Iran).^129^
- Falsification of accreditation documents (Kenya),^112,113^ or employing unlicensed staff (Indonesia).^104,120,121^
- Conflict of interest where HMOs were owned by political elites who controlled the national health insurance scheme (Nigeria).^34,108,124^
- Misuse or embezzlement of health sector funds (Mexico, Vietnam).^100,101,130^

Several measures were introduced in various countries to prevent fraud. Iran granted autonomy to teaching hospitals to manage staff motivation to reduce fraud and introduced new payment reforms to manage clinical services and balance hospital revenues.^15,131^ Mexico adjusted its regulatory framework, increasing accountability of the CNPSS and the REPSS through new accounts created at the Federal Treasury and new sanctions.^91,119^ Thailand’s NHSO introduced a global budget to augment the DRG payments and established a rigorous medical audit system.^15^

 In Indonesia, the Public Research Anti-Corruption Clearing House and the Corruption Eradication Commission was established to prevent provider fraud.^104,121^ This was supported by e-tendering for drugs and supplies, introduced to expedite contractual arrangements and reduce corruption.^120,121^ Vietnam introduced an electronic claim management system and provided smart cards for members. Ghana piloted an electronic claim submission and processing system for some providers, provided identity cards for insured members, introduced clinical audit and historical claim auditing of services provided.^126,127^ The Kenya’s MoH ensures that adequately trained medical staff conduct clinical reviews/audits in Kenya.^112,113^

 While evidence of corruption exists in Nigeria’s healthcare purchasing system, there were no descriptions of efforts to reduce it, in the literature we reviewed (Table 3). While there were no reports of corruption in China’s healthcare purchasing system, the National Health and Family Planning Commission introduced laws that prohibited corrupt practices. Providers and pharmaceuticals who were found culpable were blacklisted.

## Discussion

 Managed well, public insurance schemes, with risk and income cross-subsidisation, can provide financial protection of their members. In theory, new members join as knowledge of the scheme grows, and the benefits package can be increased as more resources become available. However, for schemes to grow beyond the mandatory enrolment of the formal sector and include the poor, the use of public funds is required; resource constraints and need for care are then at their highest, and the need for ensuring value for money even more important.^33,91,95^

 As coverage increases, a scheme needs to keep costs at an affordable level, while ensuring that OOP (ie, costs borne by members) do not escalate. Various elements of SP enable control of a schemes’ costs, such as limiting the benefit package, a cap on expenditure, using reimbursement mechanisms that enable cost control (capitation and DRGs), gate-keeping, as well as monitoring claims and minimising corruption. However, containing OOP also requires that appropriate services are provided and paid for an at appropriate rate ie, that the members do not have to seek care elsewhere,^133^ or the providers do not charge a co-payments or informal fees.^7,133^ Other elements of SP are focused on this task, such as research capacity to ensure the most appropriate services are purchased, the auditing of facilities and quality of care, as well as engagement with providers and members to understand if the scheme is meeting both of their needs.

 The schemes in Thailand and Indonesia have managed to keep OOP relatively low (11% and 18%, respectively). Both have a comprehensive benefit package, a cap on expenditure and some gate-keeping mechanism. They both have forums for systematic engagement between purchaser and providers, HTA research capacity, and have successfully reformed reimbursement mechanisms to change provider incentives. They both use international standards for accrediting facilities and conduct clinical audits of services. In sum, both have effective SP, although Indonesia has not managed to substantially include the poor (coverage is 32%) unlike Thailand (99%) where public funds subsidise membership of the poor.

 In the Vietnamese scheme, coverage is high (87%), and while benefits are limited, there is HTA capacity, a performance capitation mechanism at primary healthcare as well as a gate keeping mechanism, but FFS is used for hospital care and there is no cap on expenditure. As a result, 20% co-payments were introduced. Provider accreditation is inadequate, and there are no clinical audits. OOP are between 30%-39%. More effective cost control through a cap on expenditure and the use of DRGs might have prevented the need to introduce co-payments, and better accreditation and clinical audits might have led to better quality care, reducing the need to seek care elsewhere, both of which would have lowered OOP. Similarly, in Ghana there are limited elements of SP (FFS for hospital care, no cap on expenditure, and limited HTA capacity), and with a comprehensive benefit package, OOP is high at 50%, even though only 60% of the population is covered. In Mexico, moves toward SP that accompanied expanded coverage, did not translate to large reductions in OOPs and raised governance concerns, causing officials to revert to passive purchasing arrangements.

 If SP is to play its role in ensuring the sustainability of an insurance scheme (including fending off politically motivated demands), there needs to be considerable institutional and organisational capacity, both at the purchaser and in government.^19,134^ These include research capacity to assess health needs and which services are affordable and best value of money, the capacity to accredit facilities, monitor the quality of care and the claims submitted, and governance capacity to provide stewardship and regulation.^104,110,111^

 The literature has demonstrated that insufficient regulation leads to lack of trust between providers, purchasers, and service users, and so a failure of financial protection.^26,135^ Liu and colleagues reported that unregulated marketization of healthcare provision and inadequate financial protection by purchasers induced unhealthy competition among patients and so “red envelope” payments to secure care.^66^ Similar relationships were reported in Iran to skip long queues. Provider opportunism was fuelled by loose/non-existent regulations, disempowerment of patients to make choices,^136^ and lack of appropriate incentives for providers. For SP to ensure resources are used wisely, government regulation is also necessary.

 In their realist review of SP, Sanderson et al conclude that SP requires national government purchasers to build close, trusting relationships with providers to facilitate access to local knowledge about needs and priorities.^138^ While ‘provider decision autonomy may drive innovation and efficient resource use, it may also create scope for opportunism; interdependence [of purchasers and providers] is likely to be the best power structure to incentivise collaboration needed to drive performance improvement.’^138^ Only in two countries did we find evidence of consistent engagement between purchasers and providers.

 Ensuring patients’ rights in doing SP could be achieved through encouraging their participation in committees and boards, creating some awareness, implementing community verification of health benefits packages,^139^ ascertain population views and values.^6,15,22,91,122^ These require a degree of decentralisation and institutional purchaser capacity to engage with providers and patients that is not available in most middle-income countries.^85,113^

 In a comparison of SP in 10 European countries, Klasa et al^9^ argue that SP has not been fully implemented in any one of their case study countries; they conclude that SP is unlikely to work elsewhere and an ‘idea too perfect to exist in reality.’ Similar critiques have been raised by others.^140^ The review includes the requirement that there are sufficient providers in any location such that purchaser and patient can choose where to purchase/seek care for SP to be able to occur; we have not included this requirement, because of the idle and so wasted capacity it requires.^9^ However, there are a myriad of additional reasons why SP is hard. In practice, purchasers, often lack the data,^83,115^ expertise,^91,131^ policy capacity and negotiating power to shape an effective purchasing strategy that is focused on the quality of care and the actual needs of the population,^4^ instead of historical utilization patterns, prices and volumes.

###  Limitations 

 One of the limitations of this study is the use of heterogenous information available on the case study countries in published sources. Often information was incomplete and difficult to interpret (for example the details of the contracts and the contracting processes). Some countries may have separate HIV programmes, that are not part of the public insurance scheme, however our focus was the purchasing carried out by the insurance schemes. Interviews with key informants would have provided useful additional information and an opportunity to confirm the published sources, however, this was not possible given the resources available. Two authors were from case study countries (Ghana and Kenya) and so had a greater degree of insight, however, insights from China were sometimes limited due to certain source and government documents being available in Mandarin only.

## Conclusion

 In middle income countries, with relatively limited formal employment, managing resources well is particularly important when public funds are needed to provide cover for the poor. Schemes need to control their costs (through, for example, a cap on expenditure, capitation, DRGs, gate keeping, limiting corruption), as well as ensuring appropriate services are available and paid for at an appropriate rate which requires research capacity, audits and engagement, so that OOP do not escalate.

 While SP appears to be working well in both Thailand and Indonesia, it is only Thailand that has managed to provide a comprehensive package, include the poor, and keep OOP low. In Vietnam and Ghana, the combination of partial implementation of SP and relatively high levels of coverage is accompanied by higher levels of OOP.

 We recommend greater investment in purchaser and research capacity, and a focus on strong governance including regular engagement between purchaser, provider and citizens, that enables the building of trusting relationships. Improvements in these areas will allow countries to leverage the potential of SP more fully, thereby progressively expanding financial protection, and furthering movement towards UHC.

 The evidence from nine countries suggests that purchasing reforms, while crucial, remain difficult to enact and sustain.

## Acknowledgements

 Benjo Delarmente (a PhD Candidate) of The John Hopkins University, Baltimore, participated in the article deliberation sessions. We are grateful for his insightful contributions.

 Beryl Maritim was supported by the Consortium for Advanced Research Training in Africa (CARTA). CARTA is jointly led by the African Population and Health Research Center and the University of the Witwatersrand and funded by the Carnegie Corporation of New York (Grant No. G-19-57145), Sida (Grant No:54100113), Uppsala Monitoring Center, Norwegian Agency for Development Cooperation (Norad), and by the Wellcome Trust [reference no. 107768/Z/15/Z] and the UK Foreign, Commonwealth & Development Office, with support from the Developing Excellence in Leadership, Training and Science in Africa (DELTAS Africa) programme. The statements made and views expressed are solely the responsibility of the Fellow.

## Ethical issues

 Ethical approval was not necessary because extracted data from peer-reviewed publications.

## Competing interests

 Authors declare that they have no competing interests.

## Funding

 This project received funding from South African Chairs Initiative (SARChI) at the Centre for Health Policy (CHP), University of the Witwatersrand, Johannesburg, South Africa.

## Supplementary files

Supplementary file 1. Search Strategy.

Supplementary file 2 contains Table S1.

## References

[1] World Health Organization (WHO). The World Health Report 2000: Health Systems: Improving Performance. WHO; 2000.

[2] Kutzin J (2013). Health financing for universal coverage and health system performance: concepts and implications for policy. Bull World Health Organ.

[3] McIntyre D. Learning from Experience: Health Care Financing in Low-and Middle-Income Countries. Geneva: Global Forum for Health Research; 2007.

[4] World Health Organization (WHO). Governance for Strategic Purchasing: An Analytical Framework to Guide a Country Assessment. WHO; 2019.

[5] Honda A, Hanson K, Tangcharoensathien V, Huntington D, McIntyre D. Strategic purchasing’–definition and analytical framework used in the multi-country study. In: Strategic Purchasing in China, Indonesia and the Philippines. Geneva: World Health Organization; 2016:2.

[6] Kutzin J, Sparkes SP (2016). Health systems strengthening, universal health coverage, health security and resilience. Bull World Health Organ.

[7] RESYST. What is strategic purchasing for health? Resarchonline website. https://researchonline.lshtm.ac.uk/id/eprint/2760470/2/Purchasing%20brief.pdf. Published October 2014.

[8] Tangcharoensathien V, Limwattananon S, Patcharanarumol W, Thammatacharee J, Jongudomsuk P, Sirilak S (2015). Achieving universal health coverage goals in Thailand: the vital role of strategic purchasing. Health Policy Plan.

[9] Klasa K, Greer SL, van Ginneken E (2018). Strategic purchasing in practice: comparing ten European countries. Health Policy.

[10] Hamadeh N, Van Rompaey C, Metreau E. New World Bank Country Classifications by Income Level: 2021-2022. World Bank Blogs; 2021.

[11] Serajuddin U, Hamadeh N. New World Bank Country Classifications by Income Level: 2020-2021. World Bank Blogs; 2020. https://blogs.worldbank.org/opendata/new-world-bank-country-classifications-income-level-2020-2021. Accessed February 11, 2021.

[12] Kien VD, Van Minh H, Giang KB, Dao A, Tuan LT, Ng N (2016). Socioeconomic inequalities in catastrophic health expenditure and impoverishment associated with non-communicable diseases in urban Hanoi, Vietnam. Int J Equity Health.

[13] Nguyen H, Ivers R, Jan S, Pham C (2017). Analysis of out-of-pocket costs associated with hospitalised injuries in Vietnam. BMJ Glob Health.

[14] Onwujekwe O, Hanson K, Ichoku H, Uzochukwu B (2014). Financing incidence analysis of household out-of-pocket spending for healthcare: getting more health for money in Nigeria?. Int J Health Plann Manage.

[15] Patcharanarumol W, Panichkriangkrai W, Sommanuttaweechai A, Hanson K, Wanwong Y, Tangcharoensathien V (2018). Strategic purchasing and health system efficiency: a comparison of two financing schemes in Thailand. PLoS One.

[16] World Health Organization (WHO). The World Health Report 2003: Shaping the Future. WHO; 2003.

[17] McIntyre D, Meheus F, Røttingen JA (2017). What level of domestic government health expenditure should we aspire to for universal health coverage?. Health Econ Policy Law.

[18] World Bank. World Development Indicators. DataBank; 2021.

[19] Doshmangir L, Moshiri E, Mostafavi H, Alipouri Sakha M, Assan A (2019). Policy analysis of the Iranian Health Transformation Plan in primary healthcare. BMC Health Serv Res.

[20] Tangcharoensathien V, Patcharanarumol W, Ir P (2011). Health-financing reforms in southeast Asia: challenges in achieving universal coverage. Lancet.

[21] Gu E, Page-Jarrett I (2018). The top-level design of social health insurance reforms in China: towards universal coverage, improved benefit design, and smart payment methods. J Chin Gov.

[22] Fusheini A, Marnoch G, Gray AM (2017). Stakeholders perspectives on the success drivers in Ghana’s National Health Insurance Scheme - identifying policy translation issues. Int J Health Policy Manag.

[23] Amu H, Dickson KS, Kumi-Kyereme A, Darteh EKM (2018). Understanding variations in health insurance coverage in Ghana, Kenya, Nigeria, and Tanzania: evidence from demographic and health surveys. PLoS One.

[24] Chuma J, Maina T, Ataguba J (2012). Does the distribution of health care benefits in Kenya meet the principles of universal coverage?. BMC Public Health.

[25] Aregbeshola BS, Khan SM (2018). Determinants of catastrophic health expenditure in Nigeria. Eur J Health Econ.

[26] Kabia E, Mbau R, Oyando R (2019). “We are called the et cetera”: experiences of the poor with health financing reforms that target them in Kenya. Int J Equity Health.

[27] Kumar MB, Taegtmeyer M, Madan J (2020). How do decision-makers use evidence in community health policy and financing decisions? A qualitative study and conceptual framework in four African countries. Health Policy Plan.

[28] Agustina R, Dartanto T, Sitompul R (2019). Universal health coverage in Indonesia: concept, progress, and challenges. Lancet.

[29] Dartanto T, Halimatussadiah A, Rezki JF (2020). Why do informal sector workers not pay the premium regularly? Evidence from the National Health Insurance System in Indonesia. Appl Health Econ Health Policy.

[30] Ezeoke OP, Onwujekwe OE, Uzochukwu BS (2012). Towards universal coverage: examining costs of illness, payment, and coping strategies to different population groups in southeast Nigeria. Am J Trop Med Hyg.

[31] Ibe O, Honda A, Etiaba E, Ezumah N, Hanson K, Onwujekwe O (2017). Do beneficiaries’ views matter in healthcare purchasing decisions? Experiences from the Nigerian tax-funded health system and the formal sector social health insurance program of the National Health Insurance Scheme. Int J Equity Health.

[32] Amu H, Dickson KS, Kumi-Kyereme A, Darteh EKM (2018). Understanding variations in health insurance coverage in Ghana, Kenya, Nigeria, and Tanzania: Evidence from demographic and health surveys. PLoS One.

[33] Barimah KB, Mensah J (2013). Ghana’s National Health Insurance Scheme: insights from members, administrators and health care providers. J Health Care Poor Underserved.

[34] Etiaba E, Onwujekwe O, Honda A, Ibe O, Uzochukwu B, Hanson K (2018). Strategic purchasing for universal health coverage: examining the purchaser-provider relationship within a social health insurance scheme in Nigeria. BMJ Glob Health.

[35] Maharani A, Tampubolon G (2018). Does corporatisation improve organisational commitment? Evidence from public hospitals in Indonesia. Int J Hum ResourManag.

[36] Meemon N, Paek SC (2019). The impact of Thailand’s Universal Coverage Scheme on household catastrophic health expenditure. Asia-Pac Soc Sci Rev.

[37] Dong W, Zwi AB, Bai R, Shen C, Gao J (2021). Benefit of China’s social health insurance schemes: trend analysis and associated factors since health reform. Int J Environ Res Public Health.

[38] Mbau R, Kabia E, Honda A, Hanson K, Barasa E (2020). Examining purchasing reforms towards universal health coverage by the National Hospital Insurance Fund in Kenya. Int J Equity Health.

[39] Ebunoha GN, Ughasoro MD, Nwakoby IC, Onwujekwe OE (2020). Achieving financial risk protection through a national Social Health Insurance Programme in Nigeria: perspectives of enrollees and healthcare providers. Int J Health Plann Manage.

[40] Yang L, Sun L, Wen L (2016). Financing strategies to improve essential public health equalization and its effects in China. Int J Equity Health.

[41] Agyemang-Duah W, Peprah C, Peprah P (2019). “Let’s talk about money”: how do poor older people finance their healthcare in rural Ghana? A qualitative study. Int J Equity Health.

[42] Le Tuan P, Nam VT, Dung TC (2015). Adopting Thai Diagnosis Related Group for Vietnam universal health coverage: a case of Ba Vi district hospital. Siriraj Med J.

[43] Atuoye KN, Vercillo S, Antabe R, Galaa SZ, Luginaah I (2016). Financial sustainability versus access and quality in a challenged health system: an examination of the capitation policy debate in Ghana. Health Policy Plan.

[44] Prakongsai P, Palmer N, Uay-Trakul P, Tangcharoensathien V, Mills A (2009). The implications of benefit package design: the impact on poor Thai households of excluding renal replacement therapy. J Int Dev.

[45] Zhang H, Zhang C, Zhu S, Zhu F, Wen Y (2019). Costs of hospitalization for chronic kidney disease in Guangzhou, China. Public Adm Policy.

[46] Andoh-Adjei FX, Cornelissen D, Asante FA, Spaan E, van der Velden K (2016). Does capitation payment under national health insurance affect subscribers’ trust in their primary care provider? a cross-sectional survey of insurance subscribers in Ghana. BMC Health Serv Res.

[47] Álvarez-Hernández E, Peláez-Ballestas I, Boonen A (2012). Catastrophic health expenses and impoverishment of households of patients with rheumatoid arthritis. Reumatol Clin.

[48] Chuma J, Maina T (2012). Catastrophic health care spending and impoverishment in Kenya. BMC Health Serv Res.

[49] Agyepong IA, Nagai RA (2011). “We charge them; otherwise we cannot run the hospital” front line workers, clients and health financing policy implementation gaps in Ghana. Health Policy.

[50] Akazili J, Welaga P, Bawah A (2014). Is Ghana’s pro-poor health insurance scheme really for the poor? Evidence from Northern Ghana. BMC Health Serv Res.

[51] Le QN, Blizzard L, Si L, Giang LT, Neil AL (2020). The evolution of social health insurance in Vietnam and its role towards achieving universal health coverage. Health Policy Open.

[52] Adu-Gyamfi S, Brenya E, Amoah A (2015). National Health Insurance Scheme of Ejisu-Juaben and matters arising. Int J Soc Sci Stud.

[53] Tangcharoensathien V, Tisayaticom K, Suphanchaimat R, Vongmongkol V, Viriyathorn S, Limwattananon S (2020). Financial risk protection of Thailand’s universal health coverage: results from series of national household surveys between 1996 and 2015. Int J Equity Health.

[54] Tan SY, Qian J (2019). An unintended consequence of provider payment reform: the case of capitation grants in the National Health Insurance reform of Indonesia. Int J Health Plann Manage.

[55] Zhang G, Zhang L, Wu S, Xia X, Lu L (2016). The convergence of Chinese county government health expenditures: capitation and contribution. BMC Health Serv Res.

[56] Nketiah-Amponsah E, Alhassan RK, Ampaw S, Abuosi A (2019). Subscribers’ perception of quality of services provided by Ghana’s National Health Insurance Scheme - what are the correlates?. BMC Health Serv Res.

[57] Gao C, Xu F, Liu GG (2014). Payment reform and changes in health care in China. Soc Sci Med.

[58] Odeyemi IA, Nixon J (2013). Assessing equity in health care through the national health insurance schemes of Nigeria and Ghana: a review-based comparative analysis. Int J Equity Health.

[59] Nigenda G, Wirtz VJ, González-Robledo LM, Reich MR (2015). Evaluating the implementation of Mexico’s health reform: the case of Seguro Popular. Health Syst Reform.

[60] Cashin C, Phuong NK, Shain R, Oanh TT, Thuy NT (2015). A simple simulation model as a tool to assess alternative health care provider payment reform options in Vietnam. Glob Public Health.

[61] Tang S, Wang L, Wang H, Chin DP (2016). Access to and affordability of healthcare for TB patients in China: issues and challenges. Infect Dis Poverty.

[62] Jiang J, Lucas H, Long Q, Xin Y, Xiang L, Tang S (2019). The effect of an innovative financing and payment model for tuberculosis patients on health service utilization in China: evidence from Hubei province of China. Int J Environ Res Public Health.

[63] Mirabedini SA, Fazl Hashemi SME, Sarabi Asiabar A, Rezapour A, Azami-Aghdash S, Hosseini Amnab H (2017). Out-of-pocket and informal payments in Iran’s health care system: a systematic review and meta-analysis. Med J Islam Repub Iran.

[64] Kong XJ, Du Z, Zhao M, Yang Y, Qin Y (2011). Red envelope and doctor-patient trust: report of research on national questionnaire survey of 4000 inpatients in 10 cities (VII). Med PhilosHumanis Social Med Edit.

[65] Guo Y (2015). Personal relationships with the physicians, impression on the physicians and the urban residents’ residents’ behavior of sending the red envelopes. Chinese Health Service Management.

[66] Liu N, Bao G, He AJ (2020). Does health insurance coverage reduce informal payments? Evidence from the “red envelopes” in China. BMC Health Serv Res.

[67] García-Díaz R, Sosa-Rubí SG, Serván-Mori E, Nigenda G (2018). Welfare effects of health insurance in Mexico: the case of Seguro Popular de Salud. PLoS One.

[68] Thuong NTT, Huy TQ, Tai DA, Kien TN (2020). Impact of health insurance on health care utilisation and out-of-pocket health expenditure in Vietnam. Biomed Res Int.

[69] Duc Thanh N, Anh BTM, Thanh Hung P, Quynh Anh P, Huyen Xiem C (2021). Impact of public health insurance on out-of-pocket health expenditures of the near-poor in Vietnam. Health Serv Insights.

[70] Hidayat B. Out-of-Pocket Payments in the National Health Insurance of Indonesia: A First Year Review: Policy Brief. German National Library; 2015.

[71] Akweongo P, Aikins M, Wyss K, Salari P, Tediosi F (2021). Insured clients out-of-pocket payments for health care under the National Health Insurance Scheme in Ghana. BMC Health Serv Res.

[72] Barasa EW, Mwaura N, Rogo K, Andrawes L (2017). Extending voluntary health insurance to the informal sector: experiences and expectations of the informal sector in Kenya. Wellcome Open Res.

[73] Ebunoha GN, Ughasoro MD, Nwakoby IC, Onwujekwe OE (2020). Achieving financial risk protection through a national Social Health Insurance Programme in Nigeria: perspectives of enrollees and healthcare providers. Int J Health Plann Manage.

[74] Suphanchaimat R, Kantamaturapoj K, Pudpong N, Putthasri W, Mills A (2016). Health insurance for people with citizenship problems in Thailand: a case study of policy implementation. Health Policy Plan.

[75] Fenny AP, Asante FA, Enemark U, Hansen KS (2014). Treatment-seeking behaviour and social health insurance in Africa: the case of Ghana under the National Health Insurance Scheme. Glob J Health Sci.

[76] Phuong NK, Oanh TT, Phuong HT, Tien TV, Cashin C (2015). Assessment of systems for paying health care providers in Vietnam: implications for equity, efficiency and expanding effective health coverage. Glob Public Health.

[77] Kihuba E, Gheorghe A, Bozzani F, English M, Griffiths UK (2016). Opportunities and challenges for implementing cost accounting systems in the Kenyan health system. Glob Health Action.

[78] Munge K, Briggs AH (2014). The progressivity of health-care financing in Kenya. Health Policy Plan.

[79] Yip W, Fu H, Chen AT (2019). 10 years of health-care reform in China: progress and gaps in universal health coverage. Lancet.

[80] Zhao C, Wang C, Shen C, Wang Q (2018). China’s achievements and challenges in improving health insurance coverage. Drug DiscovTher.

[81] Sunarti S, Ghozali MT, Haris F, Rahman FF, Rahman RA (2020). Preventing fraud and deficit through the optimization of health insurance in Indonesia. Syst Rev Pharm.

[82] Zhao C, Wang C, Shen C, Wang Q (2018). Diagnosis-related group (DRG)-based case-mix funding system, a promising alternative for fee for service payment in China. Biosci Trends.

[83] Hipgrave DB, Anderson I, Sato M (2019). A rapid assessment of the political economy of health at district level, with a focus on maternal, newborn and child health, in Bangladesh, Indonesia, Nepal and the Philippines. Health Policy Plan.

[84] Limwattananon C, Limwattananon S, Tungthong J, Sirikomon K (2018). Association between a centrally reimbursed fee schedule policy and access to cataract surgery in the Universal Coverage Scheme in Thailand. JAMA Ophthalmol.

[85] Hipgrave DB, Laksmono LH, Koemarasakti GM (2018). District team problem solving as an approach to district health programme planning: a review, and survey of its status in selected districts in Indonesia. Health Policy Plan.

[86] Kazungu JS, Barasa EW (2017). Examining levels, distribution and correlates of health insurance coverage in Kenya. Trop Med Int Health.

[87] Andoh-Adjei FX, Spaan E, Asante FA, Mensah SA, van der Velden K (2016). A narrative synthesis of illustrative evidence on effects of capitation payment for primary care: lessons for Ghana and other low/middle-income countries. Ghana Med J.

[88] Doshmangir L, Bazyar M, Najafi B, Haghparast-Bidgoli H (2019). Health financing consequences of implementing health transformation plan in Iran: achievements and challenges. Int J Health Policy Manag.

[89] Martínez G (2016). [Resource allocation in the Seguro Popular program: analysis and recommendations]. Salud Publica Mex.

[90] Mathauer I, Behrendt T (2017). State budget transfers to Health Insurance to expand coverage to people outside formal sector work in Latin America. BMC Health Serv Res.

[91] González Block M, Reyes Morales H, Hurtado LC, Balandrán A, Méndez E (2020). Mexico: health system review. Health Syst Transit.

[92] Gómez-Dantés O, Frenk J (2009). Health technology assessment in Mexico. Int J Technol Assess Health Care.

[93] Lee HY, Nguyen TT, Park S, Hoang VM, Kim WH (2021). Health technology assessment development in Vietnam: a qualitative study of current progress, barriers, facilitators, and future strategies. Int J Environ Res Public Health.

[94] MacQuilkan K, Baker P, Downey L (2018). Strengthening health technology assessment systems in the global south: a comparative analysis of the HTA journeys of China, India and South Africa. Glob Health Action.

[95] Alinia C, Davoodi Lahijan J (2019). Moving toward universal health coverage: four decades of experience from the Iranian health system. Clinicoecon Outcomes Res.

[96] Ekman B, Liem NT, Duc HA, Axelson H (2008). Health insurance reform in Vietnam: a review of recent developments and future challenges. Health Policy Plan.

[97] Giménez V, Keith JR, Prior D (2019). Do healthcare financing systems influence hospital efficiency? A metafrontier approach for the case of Mexico. Health Care Manag Sci.

[98] Li Y, Ying C, Sufang G, Brant P, Bin L, Hipgrave D (2013). Evaluation, in three provinces, of the introduction and impact of China’s National Essential Medicines Scheme. Bull World Health Organ.

[99] Maharani A, Femina D, Tampubolon G (2015). Decentralization in Indonesia: lessons from cost recovery rate of district hospitals. Health Policy Plan.

[100] Mitra S, Palmer M, Mont D, Groce N (2016). Can households cope with health shocks in Vietnam?. Health Econ.

[101] Palmer MG (2014). Inequalities in universal health coverage: evidence from Vietnam. World Dev.

[102] González Block M, Reyes H, Cahuana Hurtado L, Balandrán A, Méndez E. Mexico’s health system: in longstanding pursuit of universal coverage and integration. Eur J Public Health 2020;30(Suppl 5):ckaa165.782. 10.1093/eurpub/ckaa165.782.

[103] Tangcharoensathien V, Limwattananon S, Suphanchaimat R, Patcharanarumol W, Sawaengdee K, Putthasri W (2013). Health workforce contributions to health system development: a platform for universal health coverage. Bull World Health Organ.

[104] Erniaty E, Harun H (2020). Understanding the impacts of NPM and proposed solutions to the healthcare system reforms in Indonesia: the case of BPJS. Health Policy Plan.

[105] Kazungu J, Kabia E, Munge K, Barasa E (2021). Assessing the progress and gaps in strategic health purchasing in Kenya. Wellcome Open Res.

[106] Mukhlisa MN, Pujiyanto P (2018). The effect of health insurance on institutional delivery in Indonesia. Kesmas.

[107] Pisani E, Olivier Kok M, Nugroho K (2017). Indonesia’s road to universal health coverage: a political journey. Health Policy Plan.

[108] Obikeze E, Onwujekwe O (2020). The roles of health maintenance organizations in the implementation of a social health insurance scheme in Enugu, Southeast Nigeria: a mixed-method investigation. Int J Equity Health.

[109] Tangcharoensathien V, Suphanchaimat R, Thammatacharee N, Patcharanarumol W (2012). Thailand’s universal health coverage scheme. Econ Polit Wkly.

[110] Liu J. Healthcare Quality and HIT-International Standards, China Practices. CRC Press; 2019.

[111] Kalantari AR, Jafari Sirizi M, Mehrolhassani MH, Dehnavieh R (2019). Challenges of implementation: strategic purchasing in Iran Health Insurance Organization. Int J Health Plann Manage.

[112] Smits H, Supachutikul A, Mate KS (2014). Hospital accreditation: lessons from low- and middle-income countries. Global Health.

[113] Suchman L (2018). Accrediting private providers with National Health Insurance to better serve low-income populations in Kenya and Ghana: a qualitative study. Int J Equity Health.

[114] Sieverding M, Onyango C, Suchman L (2018). Private healthcare provider experiences with social health insurance schemes: findings from a qualitative study in Ghana and Kenya. PLoS One.

[115] Luo S, Zhang K, Li B (2010). Medical informatics in China: healthcare IT trends, academic and research developments. Yearb Med Inform.

[116] Youthao S, Amornsiriphong S (2021). One Health information management: health system reforms to support social well-being in Thailand. Int J One Health.

[117] Abdulai AF, Adam F (2020). Health providers’ readiness for electronic health records adoption: a cross-sectional study of two hospitals in northern Ghana. PLoS One.

[118] Li C, Yu X, Butler JR, Yiengprugsawan V, Yu M (2011). Moving towards universal health insurance in China: performance, issues and lessons from Thailand. Soc Sci Med.

[119] González-Block M, Alarcón Irigoyen J, Figueroa Lara A, Ibarra Espinosa I, Cortés Llamas N. [The strategic purchasing of health services: a big opportunity for the National Universal Health System]. Gac Med Mex 2015;151(2):278-280. [Spanish]. 25946542

[120] Tan SY (2019). Bureaucratic autonomy and policy capacity in the implementation of capitation payment systems in primary healthcare: comparative case studies of three districts in Central Java, Indonesia. J Asian Public Policy.

[121] Khoiri A, Hidayat W, Chalidyanto D, Suhariadi F (2020). Potential of hospital fraud in the Indonesia national health insurance era (a descriptive phenomenological research). Indian J Forensic Med Toxicol.

[122] Doshmangir L, Sajadi HS, Ghiasipour M, Aboutorabi A, Gordeev VS (2020). Informal payments for inpatient health care in post-health transformation plan period: evidence from Iran. BMC Public Health.

[123] Munge K, Mulupi S, Barasa EW, Chuma J (2018). A critical analysis of purchasing arrangements in Kenya: the case of the National Hospital Insurance Fund. Int J Health Policy Manag.

[124] Lawan UM, Iliyasu Z, Daso AM (2012). Challenges to the scale-up of the Nigerian National Health Insurance Scheme: public knowledge and opinions in urban Kano, Nigeria. Ann Trop Med Public Health.

[125] Hariyanto H, Denison T, Stillman L. Understanding Health Information System Implementation in an Indonesian Primary Health Centre: A Sociotechnical Perspective. 2018. https://aisel.aisnet.org/pacis2018/219.

[126] Aikins M, Tabong PT, Salari P, Tediosi F, Asenso-Boadi FM, Akweongo P (2021). Positioning the National Health Insurance for financial sustainability and universal health coverage in Ghana: a qualitative study among key stakeholders. PLoS One.

[127] Nsiah-Boateng E, Asenso-Boadi F, Dsane-Selby L (2017). Reducing medical claims cost to Ghana’s National Health Insurance Scheme: a cross-sectional comparative assessment of the paper- and electronic-based claims reviews. BMC Health Serv Res.

[128] Joudaki H, Rashidian A, Minaei-Bidgoli B (2015). Improving fraud and abuse detection in general physician claims: a data mining study. Int J Health Policy Manag.

[129] Sajadi HS, Ehsani-Chimeh E, Majdzadeh R (2019). Universal health coverage in Iran: where we stand and how we can move forward. Med J Islam Repub Iran.

[130] Chemor Ruiz A, Ratsch AEO, Alamilla Martínez GA (2018). Mexico’s Seguro Popular: achievements and challenges. Health Syst Reform.

[131] Ehsani-Chimeh E, Sajadi HS, Majdzadeh R (2018). Iran towards universal health coverage: the role of human resources for health. Med J Islam Repub Iran.

[132] Akazili J, Gyapong J, McIntyre D (2011). Who pays for health care in Ghana?. Int J Equity Health.

[133] World Health Organization (WHO). Designing Health Financing Systems to Reduce Catastrophic Health Expenditure. WHO; 2005.

[134] Ghoddoosi-Nejad J, Jannati A, Doshmangir L, Arab-Zozani M, Imani A (2019). Stewardship as a fundamental challenge in strategic purchasing of health services: a case study of Iran. Value Health Reg Issues.

[135] Yarney L, Buabeng T, Baidoo D, Bawole JN (2016). Operationalization of the Ghanaian patients’ charter in a peri-urban public hospital: voices of healthcare workers and patients. Int J Health Policy Manag.

[136] Halvorsen K, Dihle A, Hansen C (2020). Empowerment in healthcare: a thematic synthesis and critical discussion of concept analyses of empowerment. Patient Educ Couns.

[137] Liu Y, Saltman RB (2019). Establishing appropriate agency relationships for providers in China. Inquiry.

[138] Sanderson J, Lonsdale C, Mannion R (2019). What’s needed to develop strategic purchasing in healthcare? Policy lessons from a realist review. Int J Health Policy Manag.

[139] Witter S, Bertone MP, Namakula J (2019). (How) does RBF strengthen strategic purchasing of health care? Comparing the experience of Uganda, Zimbabwe and the Democratic Republic of the Congo. Glob Health Res Policy.

[140] Paul E, Brown GW, Ridde V (2020). Misunderstandings and ambiguities in strategic purchasing in low- and middle-income countries. Int J Health Plann Manage.

