# Supplementary material for: The Experiences of Strategic Purchasing of Healthcare in Nine Middle-Income Countries: A Systematic Qualitative Review
Source: Int J Health Policy Manag. 2023 Nov 6;12:7352. doi: 10.34172/ijhpm.2023.7352 (PMC10699827; doi:10.34172/ijhpm.2023.7352)
Supplement: Supplementary file 1 — Search Strategy. [file ijhpm-12-7352-s001.pdf]

Article title: The Experiences of Strategic Purchasing of Healthcare in Nine Middle-Income Countries: A Systematic Qualitative Review

Journal name: International Journal of Health Policy and Management (IJHPM)

Authors' information: Joshua Sumankuuro[1,2,3]*, Frances Griffiths[1,4], Adam D. Koon[5], Witness Mapanga[1,6], Beryl Maritim[1,7,8], Atiya Mosam[8], Jane Goudge[1]

[1]Centre for Health Policy, School of Public Health, Faculty of Health Sciences, University of the Witwatersrand, Johannesburg, South Africa.

[2]Department of Public Policy and Management, SD Dombo University of Business and Integrated Development Studies, Wa, Ghana.

[3]School of Community Health, Charles Sturt University, Orange, NSW, Australia.

[4]Warwick Medical School, University of Warwick, Coventry, UK.

[5]Department of International Health, Johns Hopkins Bloomberg School of Public Health, Baltimore, MD, USA.

[6]School of Health Systems and Public Health, University of Pretoria, Pretoria, South Africa.

[7]Consortium for Advanced Research Training in Africa (CARTA), Nairobi, Kenya.

[8]School of Public Health, University of the Witwatersrand, Johannesburg, South Africa.

***Correspondence to:** Joshua Sumankuuro; Email: joshsumankuuro@gmail.com

Citation: Sumankuuro J, Griffiths F, Koon AD, et al. The experiences of strategic purchasing of healthcare in nine middleincome countries: a systematic qualitative review. Int J Health Policy Manag. 2023;12:7352. doi:10.34172/ijhpm.2023.7352

Supplementary file 1. Search Strategy

PubMed:

((((“Low and middle income countries” or “developing countries” OR BRICS OR BRIC OR Africa OR “north africa” or “northern Africa” OR “Africa South of the Sahara” OR “Central Africa” OR “east Africa” “Eastern Africa” or “Southern Africa” or “Western Asia” OR “central Asia” or “southeastern Asia” OR “Caribbean” OR “West Indies” OR “South America” OR “Latin America” OR “Central America” OR Afghanistan OR Albania OR Algeria OR “American Samoa” OR Angola OR "Antigua and Barbuda" OR Argentina OR Armenia OR Azerbaijan OR Bahrain OR Bangladesh OR Barbados OR Benin OR Belarus OR Belize OR Bhutan OR Bolivia OR Bosnia-Herzegovina OR Botswana OR Brazil OR Bulgaria OR Burkina Faso OR Burundi OR Cambodia OR Cameroon OR “Cape Verde” OR “Central African Republic” OR Chad OR Chile OR China OR Colombia OR Comoros OR Congo OR “Costa Rica” OR “Cote d'Ivoire” OR Croatia OR Cuba OR Cyprus OR Czechoslovakia OR “Czech

Republic” OR Slovakia OR Djibouti OR "Democratic Republic of the Congo" OR Dominica OR “Dominican Republic” OR “East Timor” OR Ecuador OR Egypt)) OR (“El Salvador” OR Eritrea OR Estonia OR Ethiopia OR Fiji OR Gabon OR Gambia OR "Georgia (Republic)" OR Ghana OR Greece OR Grenada OR Guatemala OR Guinea OR “Guinea-Bissau” OR Guam OR Guyana OR Haiti OR Honduras OR Hungary OR India OR Indonesia OR Iran OR Iraq OR Jamaica OR Jordan OR Kazakhstan OR Kenya OR Korea OR Kosovo OR Kyrgyzstan OR Laos OR Latvia OR Lebanon OR Lesotho OR Liberia OR Libya OR Lithuania OR Macedonia OR Madagascar OR Malaysia OR Malawi OR Mali OR Malta OR Mauritania OR Mauritius OR Mexico OR Micronesia OR “Middle East” OR Moldova OR Mongolia OR Montenegro OR Morocco OR Mozambique OR Myanmar OR Namibia OR Nepal OR Netherlands Antilles OR “New Caledonia” OR Nicaragua OR Niger OR Nigeria OR Oman OR Pakistan OR Palau OR Panama OR “Papua New Guinea” OR Paraguay OR Peru OR Philippines OR Poland OR Portugal OR “Puerto Rico” OR Romania OR Russia OR Rwanda OR "Saint Kitts and Nevis" OR “Saint Lucia” OR "Saint Vincent and the Grenadines" OR Samoa OR “Saudi Arabia” OR Senegal OR Serbia OR Montenegro OR Seychelles OR “Sierra Leone” OR Slovenia OR Sri Lanka OR Somalia OR “South Africa” OR Sudan OR Suriname OR Swaziland OR Syria OR Tajikistan OR Tanzania OR Thailand OR Togo OR Tonga OR "Trinidad and Tobago" OR Tunisia OR Turkey OR Turkmenistan OR Uganda OR Ukraine OR Uruguay OR USSR OR Uzbekistan OR Vanuatu OR Venezuela OR Vietnam OR Yemen OR Yugoslavia OR Zambia OR Zimbabwe))) AND ((((("Resource Allocation"[Mesh] AND purchas*)) OR "Value-Based Purchasing"[Mesh]) OR "Managed Competition"[Mesh]) OR ((Purchas*) AND (“Resource allocation” OR “Resource allocating” OR Strategic OR Tactical OR Governance or “social responsibility”))) OR ((“value based purchasing” or “managed competition” or “Value-based care” OR “Public financial management” OR “Provider payment” OR “Pay for performance” OR “Results-based financing” OR “Benefits package”)))62107:43:03#15AddSearch ((“Low and middle income countries” or “developing countries” OR BRICS OR BRIC OR Africa OR “north africa” or “northern Africa” OR “Africa South of the Sahara” OR “Central Africa” OR “east Africa” “Eastern Africa” or “Southern Africa” or “Western Asia” OR “central Asia” or “southeastern Asia” OR “Caribbean” OR “West Indies” OR “South America” OR “Latin America” OR “Central America” OR Afghanistan OR Albania OR Algeria OR “American Samoa” OR Angola OR "Antigua and Barbuda" OR Argentina OR Armenia OR Azerbaijan OR Bahrain OR Bangladesh OR Barbados OR Benin OR Belarus OR Belize OR Bhutan OR Bolivia OR Bosnia-Herzegovina OR Botswana OR Brazil OR Bulgaria OR Burkina Faso OR Burundi OR Cambodia OR Cameroon OR “Cape Verde” OR “Central African Republic” OR Chad OR Chile OR China OR Colombia OR Comoros OR Congo OR “Costa Rica” OR “Cote d'Ivoire” OR Croatia OR Cuba OR Cyprus OR Czechoslovakia OR “Czech Republic” OR Slovakia OR Djibouti OR "Democratic Republic of the Congo" OR Dominica OR “Dominican Republic” OR “East Timor” OR Ecuador OR Egypt)) OR (“El Salvador” OR Eritrea OR Estonia OR Ethiopia OR Fiji OR Gabon OR Gambia OR "Georgia (Republic)" OR Ghana OR Greece OR Grenada OR Guatemala OR Guinea OR “Guinea-Bissau” OR Guam OR Guyana OR Haiti OR Honduras OR Hungary OR India OR Indonesia OR Iran OR Iraq OR Jamaica OR Jordan OR Kazakhstan OR Kenya OR Korea OR Kosovo OR Kyrgyzstan OR Laos OR Latvia OR Lebanon OR Lesotho OR Liberia OR Libya OR Lithuania OR Macedonia OR Madagascar OR Malaysia OR Malawi

OR Mali OR Malta OR Mauritania OR Mauritius OR Mexico OR Micronesia OR “Middle East” OR Moldova OR Mongolia OR Montenegro OR Morocco OR Mozambique OR Myanmar OR Namibia OR Nepal OR Netherlands Antilles OR “New Caledonia” OR Nicaragua OR Niger OR Nigeria OR Oman OR Pakistan OR Palau OR Panama OR “Papua New Guinea” OR Paraguay OR Peru OR Philippines OR Poland OR Portugal OR “Puerto Rico” OR Romania OR Russia OR Rwanda OR "Saint Kitts and Nevis" OR “Saint Lucia” OR "Saint Vincent and the Grenadines" OR Samoa OR “Saudi Arabia” OR Senegal OR Serbia OR Montenegro OR Seychelles OR “Sierra Leone” OR Slovenia OR Sri Lanka OR Somalia OR “South Africa” OR Sudan OR Suriname OR Swaziland OR Syria OR Tajikistan OR Tanzania OR Thailand OR Togo OR Tonga OR "Trinidad and Tobago" OR Tunisia OR Turkey OR Turkmenistan OR Uganda OR Ukraine OR Uruguay OR USSR OR Uzbekistan OR Vanuatu OR Venezuela OR Vietnam OR Yemen OR Yugoslavia OR Zambia OR Zimbabwe)553235207:40:31#14AddSearch “El Salvador” OR Eritrea OR Estonia OR Ethiopia OR Fiji OR Gabon OR Gambia OR "Georgia (Republic)" OR Ghana OR Greece OR Grenada OR Guatemala OR Guinea OR “Guinea-Bissau” OR Guam OR Guyana OR Haiti OR Honduras OR Hungary OR India OR Indonesia OR Iran OR Iraq OR Jamaica OR Jordan OR Kazakhstan OR Kenya OR Korea OR Kosovo OR Kyrgyzstan OR Laos OR Latvia OR Lebanon OR Lesotho OR Liberia OR Libya OR Lithuania OR Macedonia OR Madagascar OR Malaysia OR Malawi OR Mali OR Malta OR Mauritania OR Mauritius OR Mexico OR Micronesia OR “Middle East” OR Moldova OR Mongolia OR Montenegro OR Morocco OR Mozambique OR Myanmar OR Namibia OR Nepal OR Netherlands Antilles OR “New Caledonia” OR Nicaragua OR Niger OR Nigeria OR Oman OR Pakistan OR Palau OR Panama OR “Papua New Guinea” OR Paraguay OR Peru OR Philippines OR Poland OR Portugal OR “Puerto Rico” OR Romania OR Russia OR Rwanda OR "Saint Kitts and Nevis" OR “Saint Lucia” OR "Saint Vincent and the Grenadines" OR Samoa OR “Saudi Arabia” OR Senegal OR Serbia OR Montenegro OR Seychelles OR “Sierra Leone” OR Slovenia OR Sri Lanka OR Somalia OR “South Africa” OR Sudan OR Suriname OR Swaziland OR Syria OR Tajikistan OR Tanzania OR Thailand OR Togo OR Tonga OR "Trinidad and Tobago" OR Tunisia OR Turkey OR Turkmenistan OR Uganda OR Ukraine OR Uruguay OR USSR OR Uzbekistan OR Vanuatu OR Venezuela OR Vietnam OR Yemen OR Yugoslavia OR Zambia OR Zimbabwe323935307:40:05#13AddSearch “Low and middle income countries” or “developing countries” OR BRICS OR BRIC OR Africa OR “north africa” or “northern Africa” OR “Africa South of the Sahara” OR “Central Africa” OR “east Africa” “Eastern Africa” or “Southern Africa” or “Western Asia” OR “central Asia” or “southeastern Asia” OR “Caribbean” OR “West Indies” OR “South America” OR “Latin America” OR “Central America” OR Afghanistan OR Albania OR Algeria OR “American Samoa” OR Angola OR "Antigua and Barbuda" OR Argentina OR Armenia OR Azerbaijan OR Bahrain OR Bangladesh OR Barbados OR Benin OR Belarus OR Belize OR Bhutan OR Bolivia OR Bosnia-Herzegovina OR Botswana OR Brazil OR Bulgaria OR Burkina Faso OR Burundi OR Cambodia OR Cameroon OR “Cape Verde” OR “Central African Republic” OR Chad OR Chile OR China OR Colombia OR Comoros OR Congo OR “Costa Rica” OR “Cote d'Ivoire” OR Croatia OR Cuba OR Cyprus OR Czechoslovakia OR “Czech Republic” OR Slovakia OR Djibouti OR "Democratic Republic of the Congo" OR Dominica OR “Dominican Republic” OR “East Timor” OR Ecuador OR Egypt249958307:39:40#12AddSearch ((((("Resource

Allocation"[Mesh] AND purchas*)) OR "Value-Based Purchasing"[Mesh]) OR "Managed Competition"[Mesh]) OR ((Purchas*) AND ("Resource allocation" OR "Resource allocating" OR Strategic OR Tactical OR Governance or "social responsibility"))) OR (("value based purchasing" or "managed competition" or "Value-based care" OR "Public financial management" OR "Provider payment" OR "Pay for performance" OR "Results-based financing" OR "Benefits package"))609807:39:19#11AddSearch ("value based purchasing" or "managed competition" or "Value-based care" OR "Public financial management" OR "Provider payment" OR "Pay for performance" OR "Results-based financing" OR "Benefits package")523707:38:47#10AddSearch (Purchas*) AND ("Resource allocation" OR "Resource allocating" OR Strategic OR Tactical OR Governance or "social responsibility")82707:38:04#9AddSearch "Managed Competition"[Mesh]54507:37:35#7AddSearch "Value-Based Purchasing"[Mesh]80307:37:02#5AddSearch "Resource Allocation"[Mesh] AND purchas*19007:35:47
