# Supplementary material for: The Experiences of Strategic Purchasing of Healthcare in Nine Middle-Income Countries: A Systematic Qualitative Review
Source: Int J Health Policy Manag. 2023 Nov 6;12:7352. doi: 10.34172/ijhpm.2023.7352 (PMC10699827; doi:10.34172/ijhpm.2023.7352)
Supplement: Supplementary file 2 — contains Table S1. [file ijhpm-12-7352-s002.pdf]

Article title: The Experiences of Strategic Purchasing of Healthcare in Nine Middle-Income Countries: A Systematic Qualitative Review

Journal name: International Journal of Health Policy and Management (IJHPM)

Authors' information: Joshua Sumankuuro[1,2,3]*, Frances Griffiths[1,4], Adam D. Koon[5], Witness Mapanga[1,6], Beryl Maritim[1,7,8], Atiya Mosam[8], Jane Goudge[1]

[1]Centre for Health Policy, School of Public Health, Faculty of Health Sciences, University of the Witwatersrand, Johannesburg, South Africa.

[2]Department of Public Policy and Management, SD Dombo University of Business and Integrated Development Studies, Wa, Ghana.

[3]School of Community Health, Charles Sturt University, Orange, NSW, Australia.

[4]Warwick Medical School, University of Warwick, Coventry, UK.

[5]Department of International Health, Johns Hopkins Bloomberg School of Public Health, Baltimore, MD, USA.

[6]School of Health Systems and Public Health, University of Pretoria, Pretoria, South Africa.

[7]Consortium for Advanced Research Training in Africa (CARTA), Nairobi, Kenya.

[8]School of Public Health, University of the Witwatersrand, Johannesburg, South Africa.

***Correspondence to:** Joshua Sumankuuro; Email: joshsumankuuro@gmail.com

Citation: Sumankuuro J, Griffiths F, Koon AD, et al. The experiences of strategic purchasing of healthcare in nine middleincome countries: a systematic qualitative review. Int J Health Policy Manag. 2023;12:7352. doi:10.34172/ijhpm.2023.7352

Supplementary file 2

Table S1: A description of the foundational elements required for strategic purchasing for scheme included in the analysis									
	**Iran**	**China**	**Mexico**	**Thailand**	**Vietnam**	**Indonesia**	**Ghana**	**Kenya**	**Nigeria**
**Scheme**	Social security organisation (SSO), Iran health Insurance Organisation (IHIO), Imam Khomeini Relief Foundation (IKRF)	Urban Employee's Basic Medical Insurance (UEBMI), Residents Basic Medical Insurance (RBMI)	– Seguro Popular (SP)[i]<br>– IMSS – mandatory private sector employees<br>– Institute for Social Security and Services for State Employees<br>– ISSSTE	Universal coverage scheme (UCS), Civil servants medical benefits scheme (CSMBS), social security scheme (SSS)	Social Health Insurance (SHI)	Jaminan Kesehatan Nasional (JKN)	National health insurance scheme (NHIS)	National Hospital Insurance fund (NHIF)	Formal Sector Social Health Insurance Scheme (FSSHIS)
**Purchaser**	Ministry of cooperation, labour, and social welfare (MoCLSW)	National Health Commission (NHC) purchases care for RBMI; Ministry of Human Resources and Social Security (MoHRSS) also administers the UEBMI	State's Social Protection in Health Regime (REPSS) & National Commission for Social Protection (CNPSS) purchases for IMSS/ISSSTE<br>CNPSS at state-level	-National Health Security Office (NHSO) purchases care for UCS and SSS<br>- Comptroller and accountant general purchases care for CSMBS	the Ministry of Health through the Vietnam Social Security Agency (VSS)	Badan Penyelenggara Jaminan Sosial Kesehatan (BPJS Kesehatan)	National Health Insurance Authority (NHIA)	NHIF	Health Maintenance Organisation s (HMOs)
**Cap on expenditure**	There was no overall cap on total expenditure	In 2015, 40 RMB per capita was set, with supplementary funding from local government and provincial governments based on their financial capacity and cost of health services	No cap on individual level expenditure	CSMBS: No cap; Expenditure for each member was four times higher than UCS due to FFS and lack of gatekeeper model<br>UCS: Overall cap set, and payment rates fluctuate to maintain expenditure under cap	There was no overall cap on health expenditure. However, the health insurance scheme had a cap on expenditure per health benefits per member.	Government set reimbursement price ranges for public providers based on the cost of providing services in public hospitals by level.	NHIS has no cap on expenditure	There are caps on expenditure for surgical, dialysis and oncology packages for radio and chemotherapy sessions	No mention of cap on expenditure per member
**Coverage**	96.9% (2016)	90% of rural population and 65% of urban residents (2013)	Seguro Popular – 43.5% (2015)<br>IMSS – 33% (2017)<br>ISSSTE – 7.4% (2017)	98.5% (2015)	87% (2018)	31.7% (2017)	40% (in 2014), 53-60% (2016)	16% (2016),	5% (2018)

**Eligibility**	IHIO: government employees, rural residents, the self-employed, students, disabled,<br>SSO: formal private sector employees, self-employed<br>-IKRF: the poor.	Citizens	SP-anyone not covered by formal social insurance<br>IMSS-private sector employees and dependents<br>ISSSTE-federal government employees	All residents of Thailand, including undocumented migrants	Public employees and pensioners (1992-1998)<br><br>All citizens from 1998 onwards	All residents of Indonesia are eligible to enrol on JKN.	Every person resident including undocumented migrants	Kenyan Citizens with the required documentation	Formally employed, however, Civil servants are the only people currently covered
**Contributions**	– Formal employees; 6% of min wage.<br>– Rural residents: govt pays 6% of min wage<br>– self-employed: fixed premium of which govt pays 50%.	UEBMI employer and employee contributions<br>RBMI - enrolee premiums and government subsidies	Seguro Popular is primarily from three sources (general tax revenues, family premiums, state solidarity quota), with a small contribution from high earning informal employees	UCS - Free for the poor.<br>SSS- 5% of employee income, 5% by employer and 2.75% govt<br>CSMBS - deductions from salary	Salary deductions of 2% from employee and 3% paid by the employer government tax revenues, social health insurance (SHI) funding, and OOP payments of households.<br><br>Then 20% co-payments were introduced from 1998 onwards.	– salary deductions for formal sector<br>– voluntary contributions from informal sector	Core poor, pregnant women, pensioners, children, LEAP[ii] beneficiaries, are exempted (60% of members)	– Formal: income related premium<br>– Informal Sector: a monthly premium;<br>– Free for high school students, -orphans, elderly, and disabled	5% of salary deductions for members within the public formal sector
**Benefits package**	Full: Includes all three services	Limited: Not HIV or dialysis.<br>Maternity services	Limited: Covers only maternal care including inpatient delivery, no dialysis,<br>Or HIV treatment	Full: Includes all three services	Limited: covers maternity services (after 10-12 months waiting on subscription), dialysis for revolutionary contributors and social protection group	Full: Includes all three services	Full: Part of cost of dialysis; health facility delivery; HIV/AIDS	Full: Includes all three services	Limited: Not HIV or dialysis; limited to four live births

**Gatekeeper system**	Yes	Yes, in some provinces and for some insurance schemes e.g., labour health insurance programme	Yes, however, referral network is somewhat inefficient and fragmented, which limits access to specialty care, (along with HRH limitations).	Yes, for UCS and SSS, however, CSMBS operates a non-gate keeper model.	Yes, gate keeping on a list of eligible health services to be provided	Yes	Yes	No	No
**Research**	- No research to capture population health needs, and benefits package was poorly specified<br>- some insurance organisations to violate the benefits package and provide services outside the HBP at higher premiums and out-of-pocket payments	Shanghai Clinical Research Center (SCRC), China National Development and Research Center, Duke Kunshan Global Health Research Center (GHRC), National Health and Family Planning Commission Centre for Health Statistics and Information	- National Centre for Health Technology Excellence (conducts HTA)(CENETEC)<br>- National Institute of Public Health (INSP)<br>- General Health Council defines and updates the package of high-cost interventions, certificates of health-care providers, and more recently, has developed strategies to prevent non-communicable disease.	Thai Government established several research institutions<br>– Health Intervention and Technology Assessment Programme (HITAP),<br>– Health System research institute,<br>– Thailand Research Fund.<br>– A commission on national formularies. The evidence-based approach to decision-making enabled flexibility to the operationalisation of the scheme and its alignment to population needs	– Statistics Indonesia works with the National Population and Family Planning Board and the Ministry of Health to determine population health needs and household profiles.<br>– A commission on Health Technology Assessment (HTA) and national formularies were established.	Ministry of Health conducts limited research internally, with a few publications by university academics	The Research & Development Division of the Ministry of Health has the mandate to conduct research that will inform government policy decisions.	– There is no statutory health research body<br>– NHIF conducted actuarial analysis and costing studies to determine the capitation and reimbursement rates.<br>– University academics produced limited research	- No mention of specific research organisati ons;<br>- University academics produced limited research

[i] Instituto Nacional de Salud para el Bienestar – INSABI – since January 2020. There is little evidence on the effects of the changes, hence we have focused on Segular Popular

[ii] Ghana's Livelihood Empowerment Against Poverty (LEAP) programme was established in 2008 to help alleviate poverty among the poor and vulnerable. The programme provides cash transfers to very poor people, particularly in households with orphans or vulnerable children, the elderly and people with extreme disabilities. Beneficiaries also receive free national health insurance.
